# Evolutionary patterns in squamate mitogenomes: Are selective regimes associated with fossoriality and limblessness?

**DOI:** 10.1590/1678-4685-GMB-2025-0226

**Published:** 2026-07-20

**Authors:** Anieli Guirro Pereira, Vinicius Anelli, Rubens Pasa, Karine Frehner Kavalco, Fabiano Bezerra Menegidio, Tiana Kohlsdorf

**Affiliations:** 1 Universidade de São Paulo, Faculdade de Filosofia, Ciências e Letras de Ribeirão Preto (FFCLRP), Departamento de Biologia, Laboratório de Evolução e Biologia Integrativa, Ribeirão Preto, SP, Brazil.; 2 Universidade Federal de Viçosa, Laboratório de Genética Ecológica e Evolutiva, Rio Paranaíba, MG, Brazil.; 3 Universidade de Mogi das Cruzes, Laboratório de Bioinformática e Ciências Ômicas, Mogi das Cruzes, SP, Brazil.

**Keywords:** Amphisbaena alba, limbless squamates, fossoriality, mitochondrial genomes, positive selection

## Abstract

The snakelike phenotype is characterized by limb reduction and body elongation, and independently evolved in several vertebrate lineages. This phenotype is often interpreted as adaptive to fossoriality or use of complex habitats. Limblessness and fossoriality might impose different energetic requirements for locomotion, affecting selective rates on mitochondrial genes. Previous studies identified signals of differential selection in mitochondrial genes of limbless lizards and fossorial rodents. However, it remains unclear which of these factors most intensely shapes mitochondrial genome evolution in Squamata. Amphisbaenia is a key group to answer this question, as it is one of the largest lineages of limbless and fossorial squamates. Here we report a new complete mitochondrial genome of *Amphisbaena alba* and address the relationships between limblessness and fossoriality in the evolution of mitochondrial genes in Squamata. The full length of the *A. alba* mitochondrial genome was 16,800 bp (13 protein-coding genes, 22 transfer RNAs, two ribosomal RNAs and the control region). We performed selective tests, allowing different rates for clades with limbless and fossorial species separately. Fossorial species have significant changes in selective rates in more mitochondrial genes than the limbless species, a result suggesting fossoriality as a prevalent factor shaping selective pressures on mitochondrial genes.

## Introduction

Squamata (snakes and lizards, including amphisbaenians) is the most specious order of terrestrial vertebrates, with remarkable morphological diversity, worldwide distribution, and specialization into several ecological settings (Vitt and Caldwell, 2013; Roll et al., 2017; Meiri, 2024). Perhaps the most striking and recurrent modification in the squamate *bauplan* involves evolutionary transitions from fully-limbed lacertiform morphologies to trunk-elongated and limbless forms (i.e., snakelike; Bergmann and Morinaga, 2019; Camaiti et al., 2021; Anelli et al., 2024). Limblessness evolved at least 26 times across the Squamata phylogeny (see Wiens et al., 2006; Miralles et al., 2015; Infante et al., 2018), including two specious clades, snakes (Serpentes, with almost 4200 species) and worm lizards (Amphisbaenia, with approximately 200 species), in addition to other lineages such as skinks (Scincidae) and glass lizards (Anguidae). Limbless trunk-elongated bodies provide flexibility and maneuverability during locomotion in complex environments (Van Damme and Vanhooydonck, 2002). While lacertiform lizards locomote using coordinated movements of their four limbs, the snakelike species move using particular undulation strategies along their axial skeleton (Bergmann and Morinaga, 2019; Bergmann *et al*., 2020). In this context, snakelike locomotion involves specific anatomical and physiological patterns (Gasc, 1981; Navas et al., 2004). The reduction of limb musculature and reliance on axial undulatory locomotion may impact metabolic demands, potentially imposing additional energetic costs (Escalante et al., 2021; Wu et al., 2022) and, consequently, driving shifts in selective pressures acting during the evolution of mitochondrial genes (Wang et al., 2021; Wu *et al*., 2022). Mitochondrial genes contribute for the biogenesis of the ATP-synthesizing machinery (Chen and Butow, 2005), and previous studies reported accelerated evolution in certain mitochondrial genes (e.g., *ATP6* and *ND2*) among limbless squamates (e.g., Wang *et al*., 2021; Wu *et al*., 2022). Current literature encompasses a growing body of evidence suggesting that changes in locomotor behavior may also imprint detectable signatures in the mitochondrial genome, as reported for bats (Shen et al., 2010), birds (Shen *et al*., 2009), and fishes (Sun et al., 2011; Consuegra et al., 2015; Sebastian et al. 2020; Baltazar-Soares et al., 2021). 

The evolution of limblessness in squamates is often associated with the occupation of subterranean niches and the emergence of taxa successfully adapted to a fossorial lifestyle, characterized by specialized features that enhance burrowing and movement underground (see Navas et al., 2004; Miralles et al., 2015; Anelli et al., 2024). Adaptations to subterranean environments entail notable physiological challenges, given the substantial energy expenditure associated with burrowing in low-oxygen conditions (Navas *et al*., 2004). Several studies demonstrated directional positive selection in mitochondrial genes among lineages that live underground (frequently in *CYTB*, but also in other mitochondrial genes; see Silva et al., 2009; Tomasco and Lessa, 2011, 2014; Gan et al., 2018; Tavares and Seuánez, 2018), but effects of these two features - fossoriality and limblessness - have not been evaluated together.

Molecular signatures in protein-coding mitochondrial genes of squamates represent a fascinating topic because mitogenomic patterns may reveal traces of evolutionary processes associated with fossoriality, limblessness, or a combined effect of both. The recurrent and convergent evolution of fossorial and limbless forms within Squamata constitutes a promising framework for exploring how locomotor adaptations and ecological specialization can shape the evolution of mitochondrial genomes. Similar to other vertebrates, the mitochondrial genomes of squamates correspond to the characteristic circular double-stranded DNA molecule that usually ranges from 16 to 19 kb in length. The strands are distinguished by their nucleotide composition: Heavy (H-strand) is guanine-rich, whereas Light (L-strand) is rich in cytosine (Chinnery and Hudson, 2013). Squamate genomes maintain a conserved genetic composition that comprises 13 protein-coding genes, 22 tRNAs, two rRNAs and a non-coding control region (Boore, 1999; Pereira, 2000). All 13 proteins encoded by the mitochondria are essential elements of the ATP-synthesizing machinery already experiencing significant positive selection, in collaboration with 80 additional proteins encoded by nuclear genes (Chen and Butow, 2005; Sun et al., 2011; Nabholz et al., 2012). Together, these proteins constitute the oxidative phosphorylation (OXPHOS) machinery (Wallace, 2005). Despite its importance in metabolism, we lack studies about the evolution of mitochondrial genes in fossorial and limbless squamates, and the question of which effects prevail during the evolution of mitochondrial genomes - if fossoriality or limblessness - remains unexplored in this clade.

Amphisbaenia is a key group to answer this type of question. These animals are limbless (except for the limb-reduced genus *Bipes*) and adapted to a fossorial lifestyle, being the most-specious squamate clade composed exclusively by snakelike and fossorial species (Kearney, 2003; Kearney and Stuart, 2004). Amphisbaenians are distributed across the Americas including the Caribbean, and also in Africa, Europe and Eastern Asia (Gans, 1990; Hembree, 2006). Their locomotor behavior has been studied since the 1960s (Gans, 1968), and they employ different undulatory mechanisms to propel their bodies through the substrate with their heads, a locomotion often referred to as ‘head-first burrowing’ (Navas et al., 2004; Hohl et al., 2014). Despite the long-standing interest in the morphology and biomechanics of amphisbaenians, genomic data remain scarce for this clade. To date, only eight complete mitochondrial genomes have been sequenced for the group, including a single species of the genus *Amphisbaena* (*A. schmidti*). Only one species has a complete nuclear genome available: *Rhineura floridana*. The scarcity of genomes available for amphisbaenians contrasts with the increased sequencing of genomes for snakes (mitochondrial genomes available for 137 species and complete nuclear genomes for 167 species) and other limb-reduced lizards in the past decade. Here, we report a new complete mitochondrial genome of *Amphisbaena alba*, a species with a neotropical distribution. We combined this new genome with other mitogenomes available for limbless and fossorial squamate species to investigate the interplay of selective pressures related to limblessness and fossoriality during the evolution of protein-coding mitochondrial genes in Squamata. These genetic signatures can provide insights into how terrestrial vertebrate species have adapted to diverse environmental niches, and also enable evaluation of the influence of morphological, locomotor and ecological diversification on the evolution of mitochondrial genes and cellular respiration.

## Material and Methods

### *Amphisbaena alba* mitochondrial genome assemblage: specimen collection, DNA extraction and sequencing

A specimen of *Amphisbaena alba* was collected at the University of São Paulo campus in Ribeirão Preto, Brazil (21.164286° S, 47.860122° W), and deposited in the Herpetological Collection of Ribeirão Preto (CHRP-USP, voucher CHRP 5531; collected on March 19, 2020 under Sisbio-Brazil Permit #33335-2). A liver tissue sample was extracted and stored at -80°C. Total genomic DNA was isolated using the DNeasy Tissue Kit from Qiagen (catalogue number 69504) following the manufacturer instructions. The DNA was subsequently sequenced across two lanes on an Illumina NovaSeq platform at a sequencing facility, generating a total of 253.7 million clusters (507.5 million paired-end reads, 150 bp each), resulting in an estimated genome coverage of approximately 50.7x. The sequencing quality was high, with 94.3% of reads achieving Q20 (99% accuracy) and 87.5% achieving Q30 (99.9% accuracy). After filtering low-quality reads, the complete mitochondrial genome was assembled *de novo* from the clean data using GetOrganelle v.1.7.7.0 (Jin et al., 2020). The genome assembly was performed using the animal mitochondrial workflow (-F animal_mt) with modified parameters (-r 40 -k 21, 45, 65, 85, 105 -w 50). The resulting mitogenome was annotated using the MitoAnnotator tool (Iwasaki et al., 2013) on the MitoFish server (http://mitofish.aori.u-tokyo.ac.jp). A subsequent BLAST search performed in the NCBI database (https://blast.ncbi.nlm.nih.gov/) revealed a close genetic relationship between *A. alba* and *A. schmidti*, the only other congeneric species with a mitochondrial genome available in the database.

### Sequences for other squamate species and ecological and morphological classification

We downloaded all 308 mitochondrial genomes available for squamate species in the GenBank, excluding Gekkota and Dibamidae (see Table S1). We did not include the family Dibamidae, the earliest-diverging lineage within Squamata, due to the lack of information for this group, as currently no complete mitochondrial genomes are available for several species of Dibamidae in the NCBI database. A complete genome is available for *Dibamus cf. smithi*, which allows retrieving the mitochondrial genes of this species, but the clade is entirely limbless and therefore lacks comparable limbed species for meaningful within-group comparisons. The other lineage not included was Gekkota, because only one genome available represents a limbless fossorial species (*Aprasia parapulchella*), which would be paired with very distantly-related species and substantially increase the genetic divergence among the sampled taxa. Given that we already had assembled a very robust dataset compatible with the computational constraints of the analyses, and in order to avoid unnecessary phylogenetic heterogeneity and imbalance in taxon representation, we opted to not include Dibamidae and Gekkota and focus our analyses on the remaining squamate lineages, which match the premises established for our study.

In our analyses, we used two published phylogenies available for Squamata (Tonini et al., 2016; Title et al., 2024), which are based on mitochondrial and nuclear data. While Tonini *et al*. (2016) constructed a time-calibrated, fully sampled squamate phylogeny using taxonomic constraints, Title *et al*. (2024) generated a time-calibrated squamate phylogeny based on a phylogenomic backbone that includes only species with molecular data. We matched the species for which mitochondrial genomes are available to the corresponding lineages represented in each phylogeny. 

To ensure consistency, some species names in the trees were updated based on GARD (Global Assessment Reptile Distribution, version 1.7; Roll et al., 2017; Caetano et al., 2022) and the Reptile Database (http://www.reptile-database.org; Uetz *et al*., 2025). Species that were not represented in the phylogenies were excluded. In genera represented by several species, we limited sampling to three species that represented the most-distantly related species within the genus. The analyses of selection regimes impose computational limits due to the exponential increase in model complexity and likelihood calculations, so we analyzed a subset of the total species database we assembled, maintaining all lineages representing fossoriality and/or limblessness. Each focal species was paired with an equal number of closely-related species limbed and non-fossorial, controlling for unequal taxon sampling. Sea snakes were excluded from the dataset to avoid confusion in the ecological classification regarding fossoriality. The final dataset comprised 55 species, and we pruned both phylogenies to match that reduced dataset. Given that both topologies remained identical when using the 55 species, we performed all analyses using the hypothesis published by Title et al. (2024).

**Figure 1 -:**
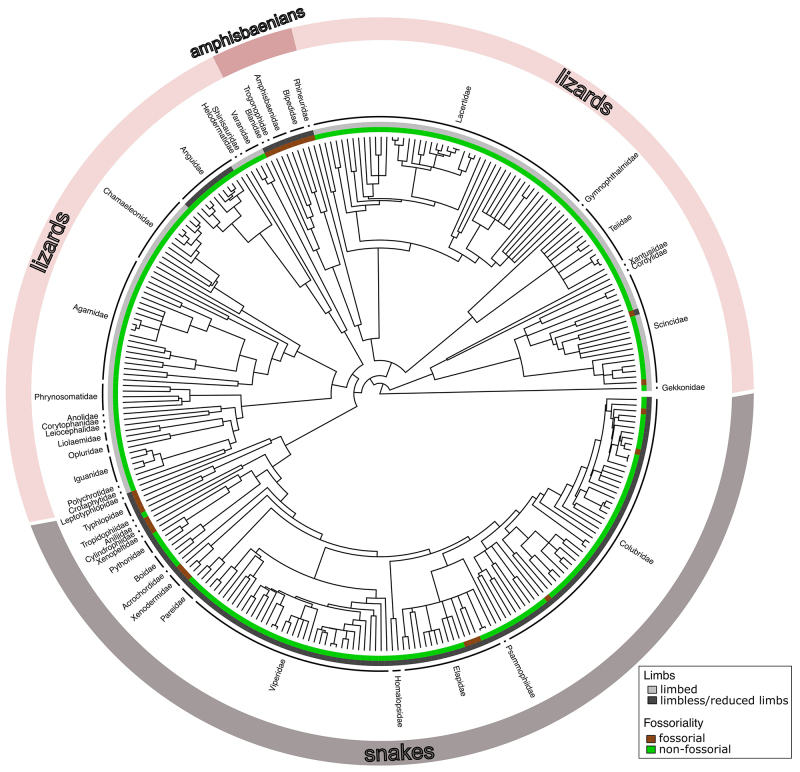
Phylogeny of Squamata from Title et al. (2024) including 308 species from 43 families. The most external ring indicates the main lineages (snakes, lizards, and amphisbaenians); inner rings represent families.

Finally, we classified the 55 species from the reduced dataset according to their fossoriality and limblessness (Figure 1), following Cyriac and Kodandaramaiah (2018). For species not included in that study (Cyriac and Kodandaramaiah, 2018), we obtained information from the IUCN Red List of Threatened Species and additional literature (e.g., Bars-Closel et al., 2017; Anelli et al., 2024). For the analyses, we considered two classifications: one distinguishing between fossorial and non-fossorial species, and another separating fully-limbed from limbless (or limb-reduced) species. 

### Mitogenome structures

To compare the mitogenome structure of *Amphisbaena alba* with that of other amphisbaenians, we analyzed all mitochondrial genomes available for the group in our dataset using the MitoFish server. The analyzed species included *Amphisbaena schmidti*, *Bipes biporus*, *Bipes canaliculatus*, *Bipes tridactylus*, *Diplometopon zarudnyi*, *Geocalamus acutus*, and *Rhineura floridana* (from Macey et al., 2004), as well as *Blanus cinereus* (from Albert et al., 2009). In addition, we included *Lacerta agilis* (NC_021766.1) as an outgroup. The MitoFish analysis provided genome sizes, GC content, and gene order for all species.

### Correlations between mitogenomes, fossoriality and limblessness

We first generated independent alignments of the 13 protein-coding genes (PCGs) using MAFFT v7.526 (Katoh and Standley, 2013). The resulting alignments were subsequently inspected and manually trimmed in SeaView v5.0.5 (Gouy et al., 2010). To improve alignment accuracy and minimize potential frameshifts or misalignments in poorly conserved regions, sequences were translated into amino acids and realigned at the protein level using SeaView (Gouy *et al*., 2010).

Associations between limblessness and fossoriality and shifts in evolutionary rates at specific amino-acid sites were tested using TraitRateProp (Karin et al., 2017). Species exhibiting the foreground trait were coded as ‘1’, whereas all other species were coded as ‘0’. The analyses were performed on nucleotide sequences translated into amino-acid sequences.

### Analyses of selection regimes

Considering that the substitutional saturation can potentially bias molecular evolutionary inferences (especially in rapidly-evolving mitochondrial genes, which may evolve up to 25 times faster than nuclear loci in amphibians and reptiles; see Lynch et al. 2006), first we evaluated saturation levels to ensure the robustness of selective estimates. We assessed substitution saturation using the iss (index of substitution saturation) statistics implemented in DAMBE (Xia and Xie, 2001; Xia *et al*., 2003; Xia and Lemey, 2009). The proportion of invariant sites required for the analysis was also estimated using DAMBE.

Then, we tested the effects of the limbless morphology and the fossorial habit in selective regimes acting on the evolution of mitogenomes in Squamata using the ete-evol module of the ETE Toolkit Python environment (v. 3.0.0b35; Huerta-Cepas et al., 2016), which implements the CODEML program from the PAML package (Yang, 2007). As the ETE Toolkit estimates branch-specific ω values, the ETE annotations on the phylogeny were consolidated into uniform category markings. A subsequent analysis was then performed using CODEML in PAML v4.10.9, in which a single ω value was estimated for all species sharing the focal trait (limbless, fossorial, or limbless-fossorial). The analyses were performed for each gene separately as well as for the concatenated dataset of all genes together (hereafter referred to as “ALL”). We fixed the topology from Title et al. (2024) without branch lengths, as these were subsequently estimated during the PAML analyses.

The ω ratio, calculated as the ratio of non-synonymous to synonymous substitution rates (dN/dS), estimates the type of selection acting on protein-coding sequences, where ω < 1 means purifying selection, ω = 1 corresponds to neutral evolution, and ω > 1 is interpreted as positive selection (Yang, 2007). We first ran the one-rate model (M0), in which a single ω value is estimated for the entire phylogeny. We also ran the M0 model with ω fixed at 1 to test whether the gene evolved under non-neutral selection. To specifically assess whether only the foreground branches evolved under non-neutral selection (ω ≠ 1), we used the b_neut model (where ω_foreground = 1 and ω_background is estimated). Finally, we applied the b_free model, which allows independent estimation of ω for both foreground and background branches (see Yang and Nielsen, 2002; Huerta-Cepas et al., 2016), to test for differences in selective regimes between these lineages.

To confirm our results, particularly for potentially saturated genes, we also performed branch-site model analyses, as previous studies suggest that this type of analysis is robust even for saturated genes (Gharib and Robinson-Rechavi, 2013). The branch-site model tests foreground branches and also classifies sites into two or three categories of different ω values. We generated input files for PAML using ETE3, and labeled the trees so that the same parameters were estimated for the foreground lineages. We then ran analyses in PAML, testing the bsC versus M1 and the bsD versus M3 models. In the bsC-M1 comparison, the null model (M1) assumes two site classes with ω ≤ 1 across all branches, whereas the alternative branch-site model (bsC) allows an additional class of sites on the foreground branches with ω > 1, thus explicitly testing for positive selection on a subset of sites in the target lineages (Yang and Nielsen, 2002). In the bsD-M3 comparison, the null model (M3) allows several site classes with different ω values shared across all branches, while the branch-site model (bsD) allows the foreground branches to have their own ω distribution, with an extra class of sites potentially evolving with ω > 1, thereby providing a more flexible test of lineage-specific shifts in selective regimes (Yang and Nielsen, 2002; Bielawski and Yang, 2004).

Model comparisons were performed using likelihood ratio tests (LRTs), in which twice the difference in log-likelihoods (2ΔlnL) between nested models was compared to a chi-square (χ²) distribution, with degrees of freedom corresponding to the difference in the number of estimated parameters (np) between models (see Yang, 2007). All calculations were carried out using Google Sheets formulas. LRTs were performed to compare a neutral model (M0, ω = 1) with the M0 non-neutral model (M0). We also compared the foreground non-neutral model (b_neut, where ω_foreground_ = 1) with an alternative model (b_free) that allows ω_foreground_ ≠ 1 and ω_foreground_ ≠ ω_background_ (see Yang and Nielsen, 2002; Huerta-Cepas et al., 2016). Next, we tested whether ω values differed between the foreground and the remaining branches (background) by comparing the fit of the b_free model with the fit of a one-rate model (M0), in which a single ω value is estimated for the entire phylogeny. These analyses were performed on three datasets: (1) limbless *versus* limbed lineages, (2) fossorial *versus* non-fossorial lineages, and (3) only limbless *versus* only fossorial *versus* lineages that are both limbless and fossorial. 

Subsequently, we applied the RELAX method (Wertheim et al., 2015), implemented in HyPhy (Pond et al., 2005, 2020), to assess shifts in the intensity of selection (relaxation or intensification) in the foreground lineages, using the default parameters. RELAX compares a null codon model, consisting of three ω classes across the phylogeny, with an alternative model that allows relaxed or intensified selection (see Wertheim *et al*., 2015). This involves the parameter k, which quantifies the selection intensity relative to the reference branches, where k > 1 corresponds to intensified selection relative to the background and k < 1 indicates relaxed selection relative to the background. LRTs were then performed to compare the fit between the null and alternative models (Wertheim *et al*., 2015). Because RELAX only accepts two categories, analyses were performed for (1) limbless *versus* limbed lineages and (2) fossorial *versus* non-fossorial lineages. Results were visualized and interpreted using HyPhy Vision (Pond *et al*., 2020).

For the PAML analyses, we applied the same mathematical basis used in RELAX to derive a descriptive log-transformed scaling parameter [ln(ω_foreground_) / ln(ω_background_)], which we hereafter refer to as *R* (a ratio analogous to the selection intensity parameter k in RELAX). In RELAX, this transformation is applied to three ω categories corresponding to different site classes. However, it was necessary to simplify our *R* estimates because PAML provides a single overall ω estimate, rather than multiple ω categories. The parameter *R* was calculated to facilitate graphical visualization of foreground ω estimates relative to the background. Like the k parameter, *R* quantifies the selection intensity relative to the background branches, where k > 1 indicates intensified selection and k < 1 corresponds to relaxed selection. For the PAML branch-site analyses, the R value was calculated for the third site class, which corresponds to sites that differ between background and foreground lineages. Given the substantially increased number of parameters in this analysis, we avoided performing separate analyses for each foreground group, as this would further inflate model parameterization and complexity, potentially reducing computational tractability and the reliability of parameter estimation.

## Results

### Organization and characteristics of the mitochondrial genome of *Amphisbaena alba*

**Figure 2 -:**
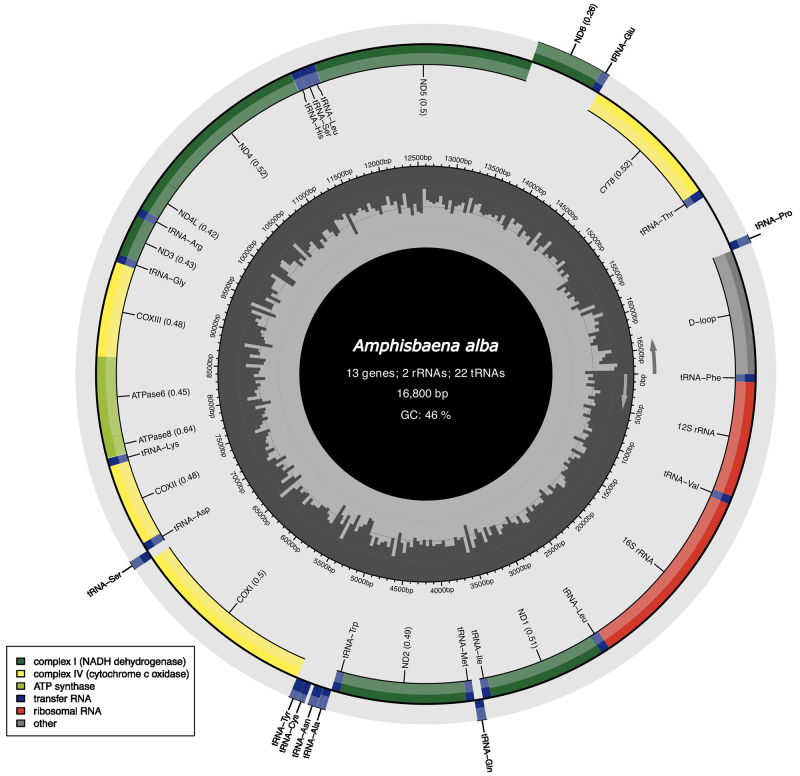
Circular visualization map of the complete mitochondrial genome of *Amphisbaena alba* created by Chloroplot. The external color circle shows the gene map (PCGs, rRNAs, tRNAs); in the innermost circle, dark gray lines represent higher GC% *per* 5bp of the mitogenome; the darker the lines are, the higher is their GC% content.

The total length of the mitochondrial genome of *A. alba* was established in 16,800 base pairs (GenBank accession number: PZ210514), with a GC content of 46% (Figure 2). Genome size and associated information for the data retrieved for other amphisbaenians and other squamate clades are reported in the Table S1 and Figures S1 and S2. These genomes have the typical circular organization observed in vertebrates, and contain 13 protein-coding genes (*ND1-6*, *ND4L*, *COI-III*, *CYTB*, *ATP6*, and *ATP8*), 22 transfer RNAs, two ribosomal RNA genes (*12s* rRNA and *16s* rRNA), and a control region (D-loop). Among these, *ND6* and eight tRNAs (tRNA^Gln^, tRNA^Ala^, tRNA^Asn^, tRNA^Cys^, tRNA^Tyr^, tRNA^Ser^, tRNA^Glu^, and tRNA^Pro^) are encoded on the light strand, while the remaining genes are encoded on the heavy strand.

*Correlations between mitogenomes, fossoriality and limblessness*

TraitRateProp analyses suggested associations between shifts in evolutionary rates and limblessness (p-values < 0.001) and fossoriality (p-values < 0.041) (Table 1). However, we found no evidence of specific amino acid substitutions consistently associated with any of these traits (empirical posterior Bayes < 1; Figure S3).

**Table 1 -:** Results of RateTraitProp analyses testing for trait-associated rate variation in mitochondrial genes of squamate species. For each gene, comparisons between the alternative model (trait-dependent rates) and the null homogeneous-rate model (M0) are shown for limbless and fossorial lineages. Reported values include the log-likelihood of the alternative model (lnL_alt), the log-likelihood of the null model (lnL_M0), the likelihood ratio test statistic (D = 2ΔlnL), and the corresponding p-value (p). Significant p-values (p < 0.05) indicate support for trait-associated shifts in evolutionary rates.

Gene	Limbless				Fossorial			
	lnL alt	lnL M0	D	p	lnL alt	lnL M0	D	p
ATP6	-10359.5	-10402.8	86.6	<0.001	-10411.6	-10413.7	4.2	0.040
ATP8	-4744.7	-4762.7	36.0	<0.001	-4770.0	-4773.6	7.2	0.007
COX1	-8933.2	-8983.0	99.5	<0.001	-8980.9	-8993.9	25.9	<0.001
COX2	-7448.8	-7503.6	109.7	<0.001	-7502.4	-7514.5	24.2	<0.001
COX3	-7154.1	-7198.5	88.9	<0.001	-7206.3	-7209.4	6.3	0.012
CYTB	-13562.4	-13640.0	155.2	<0.001	-13641.3	-13650.9	19.2	<0.001
ND1	-10667.9	-10685.2	34.6	<0.001	-10685.5	-10696.1	21.2	<0.001
ND2	-19329.5	-19369.5	80.0	<0.001	-19375.3	-19380.4	10.2	0.001
ND3	-5294.6	-5318.4	47.7	<0.001	-5325.8	-5329.3	6.9	0.009
ND4	-20759.2	-20810.0	101.6	<0.001	-20805.7	-20820.9	30.4	<0.001
ND4L	-5269.3	-5286.1	33.6	<0.001	-5290.7	-5297.0	12.5	<0.001
ND5	-32490.8	-32726.3	471.0	<0.001	-31461.2	-32737.1	2551.8	<0.001
ND6	-11850.8	-11960.0	218.4	<0.001	-11943.5	-11970.9	54.8	<0.001

*Selection regimes in limbless and fossorial squamates*

For most species, the analyses of substitution saturation indicated values of the index of substitution saturation (iss) that were significantly lower than the corresponding critical iss.c thresholds under both symmetrical and asymmetrical topology assumptions, indicating little substitution saturation. However, two genes (*ATP8* and *ND6*) exhibited iss values exceeding the critical iss.c under both symmetrical and asymmetrical topologies for most species (Table S2). When considering only asymmetrical topologies, a larger number of genes exhibited iss values exceeding the critical iss.c: *ND2, ND3, ND4, ND4L*, and *ND5*. 

**Figure 3 -:**
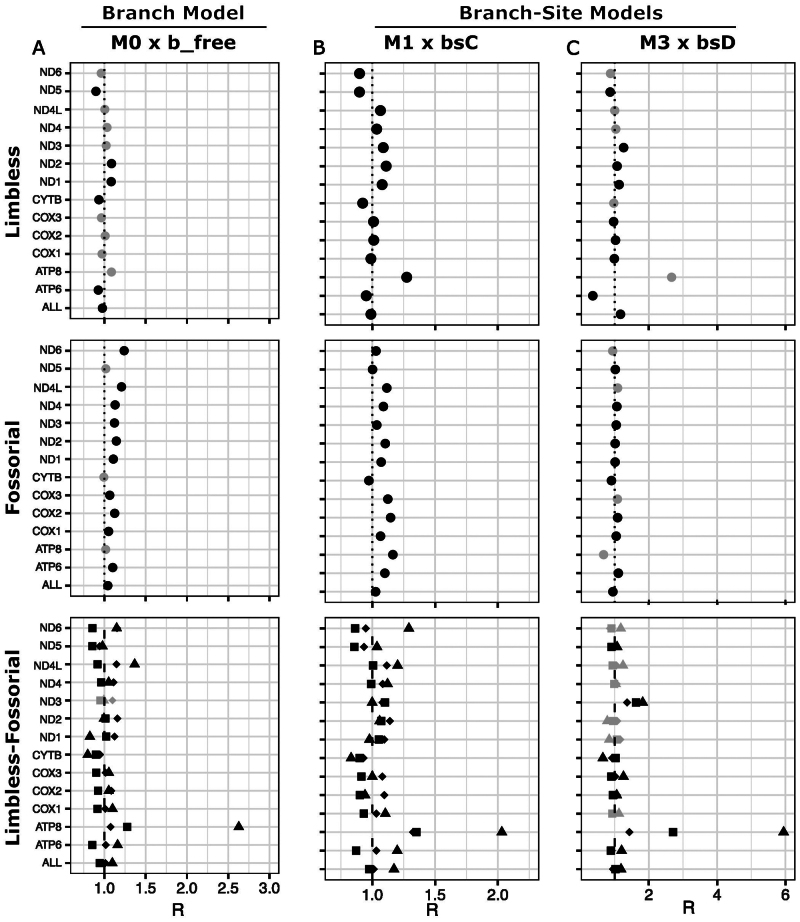
Results of PAML analyses. Log-ratio of selective rates [R = ln(ω_foreground) / ln(ω_background)] comparing ω values between foreground and background lineages. Grey circles indicate the genes with non-significant results (p > 0.05). (A) Comparison between branch models (M0 *vs* b_free); (B) branch-site model comparison between M1 and bsC; (C) branch-site model comparison between M3 and bsD. The first row corresponds to limbless species, the second to fossorial species, and the third separates species into limbless (squares), fossorial (triangles), and limbless-fossorial (diamonds) groups.

In the CodeML/PAML branch model analyses (b_free *vs* M0), when all genes were analyzed together, selection ratios (ω) differed significantly between limbless and the background lineages (p = 0.0025) and between fossorial and the background lineages (p < 0.001), as illustrated in Figure 3 A . Notably, limbless lineages exhibited higher ω values than the background, indicating relaxed selection (ω_background_ = 0.0782; ω_foreground_ = 0.0831; R = 0.9761), whereas fossorial lineages showed lower ω values, consistent with intensified selection (ω_background_ = 0.0844; ω_foreground_ = 0.0761; R = 1.0417). However, in gene-by-gene analyses, we identified higher ω values (i.e., weaker purifying selection) in limbless species than in limbed species for the genes *ATP6, CYTB,* and *ND5* (0.897 < R < 0.977; p-values for these genes lower than 0.032), whereas lower ω values (i.e., stronger purifying selection) were observed in the genes *ND1* and *ND2* in limbless lineages (1.082 < R < 1.087; p-values for these genes lower than 0.006). We did not identify significant differences between these two groups in the remaining genes (p-values for these genes larger than 0.140). In contrast, in comparisons addressing fossoriality, we identified strong evidence for purifying selection in the foreground lineages (fossorial) in nearly all genes (*ATP6, COX1, COX2, COX3, ND1, ND2, ND3, ND4, ND4L*, and *ND6*; 1.050 < R < 1.240; p-values for these genes lower than 0.026).These results are detailed in Table S3. 

When lineages were partitioned into four groups (background, fossorial, limbless, and limbless-fossorial), the differences remained highly significant when all genes were analyzed together (p < 0.0001; see Table S3). Consistently, the group exhibiting the strongest selective constraint (i.e., the lowest ω value) was the fossorial-only group, which in our dataset is represented solely by *Plestiodon egregious* (ω = 0.062; R = 1.096), followed by limbless-fossorial species (ω = 0.077; R = 1.012). In contrast, we found more evidence for relaxed selection (R = 0.944) in limbless species (ω = 0.091) compared to the background (ω = 0.079). When analyses were performed on individual genes, almost all tests indicated significant differences in ω values among groups (p < 0.024), except for *ND3* (p *=* 0.120). Notably, we found evidence of intensification of selection in nearly all genes in the limbless-fossorial group (1.010 < R < 1.158), except for *CYTB* and *ND5* (R < 0.949). In the fossorial-only group, analyses suggested intensification of selection in eight genes (*ATP6, ATP8, COX1, COX2, COX3, ND4, ND4L,* and *ND6*; 1.051 < R < 2.628), whereas this type of selection was identified in only three genes (*ATP8, ND1,* and *ND2;* 1.013 < R < 1.277) in the limbless-only group.

When limbless groups were analyzed separately using concatenated genes, we detected significant differences among clades in the ω values (p < 0.0001; Table S4). The strongest signal of purifying selection was identified in *Isopachys gyldenstolpei* (ω = 0.050; R = 1.175), followed by the clade Amphisbaenia (ω = 0.074; R = 1.024), whereas snakes (ω = 0.088; R = 0.955) and species of the Anguinae subfamily (ω = 0.083; R = 0.976) exhibited ω values closer to neutrality than those associated to the background (ω = 0.079). In the analyses focusing on fossoriality, we only detected ω values larger than those of the background (ω = 0.085) in the genera *Achalinus* (ω = 0.096; R = 0.948) and *Calamaria* (ω = 0.094; R = 0.958), whereas all other fossorial clades exhibited stronger signal of purifying selection than the non-fossorial lineages. The lowest ω value, which indicates stronger selection, was observed in the species *Isopachys gyldenstolpei* (ω = 0.0496; R = 1.217), followed by *Xenopeltis unicolor* (ω = 0.060; R = 1.142), *Plestiodon egregius* (ω = 0.061; R = 1.135), *Anilius scytale* (ω = 0.067; R = 1.096), *Cylindrophis ruffus* (ω = 0.069; R = 1.083), the clades Amphisbaenia (ω = 0.074; R = 1.056) and Scolecophidia (ω = 0.077; R = 1.036), and the snake *Micrurus fulvius* (ω = 0.078; R = 1.033).

The results from analyses using the branch-site model were largely consistent when species were divided into four groups (limbless-only, fossorial-only, and limbless-fossorial and background; see Figures 3 B and 3C and Table S5). All p-values from comparisons between the null model (M1) and the alternative model bsC (which estimates different ω values for the groups) were statistically significant (p < 0.05). Here we focus on the results from the comparison between null model M3 and the alternative model bsD (different ω values for the groups); only comparisons related to the genes *COX1*, *ND1*, *ND2*, *ND4L*, and *ND5* were not statistically different (p > 0.0578). Among the other genes, four (*ATP8*, *CYTB*, *ND3*, and *ND5*) exhibited a subset of sites with stronger signal for selective regimes in limbless lineages than in the background. In fossorial lineages, we detected evidence of sites evolving under stronger selection in seven genes (*ATP6*, *ATP8*, *COX2*, *COX3*, *ND3*, *ND4*, and *ND5*), whereas in limbless-fossorial lineages this pattern was detected in five genes (*ATP8*, *COX2*, *COX3*, *ND3*, and *ND4*). In the concatenated analysis, we identified higher ω values in limbless-only and fossorial-only groups (R = 1.023 and R = 1.185, respectively), whereas rates associated with the limbless-fossorial group were more nearly neutral (R = 0.951).

The RELAX analyses revealed that limbless species exhibited lower ω values in *ATP6*, *COX1*, *COX2*, *CYTB*, and *ND5*, suggesting stronger purifying selection acting on these mitochondrial genes. Similarly, fossorial species showed decreased ω values in *ATP6, ATP8, CYTB,* and *ND5* (Figure S4 and Table S6).

## Discussion

In this study, we combined a newly generated mitochondrial genome of *Amphisbaena alba* with other genomes available for Squamata and applied comparative analyses to evaluate how limblessness and fossoriality shaped mitochondrial genome evolution in the clade. As a result, we identified significant changes in selective rates in a larger number of mitochondrial genes associated with fossoriality, when compared to limblessness, suggesting this ecological shift as a prevalent factor shaping selective pressures on mitochondrial genes. The mitochondrial genome of *A. alba* that we sequenced has 16,800 base pairs and a GC content of 46%, with the typical circular organization observed in reptiles and the same gene arrangement reported for other squamates. Based on genome sizes available in GenBank, the assembled mitogenome falls within the range reported for other amphisbaenians (16.2 kb in *Bipes canaliculatus* to 17.4 kb in *Amphisbaena schmidti*) and other squamates (12.6 kb in *Phrynocephalus maculatus* to 26.3 kb in *Hydrophis ornatus*), although these values may be slightly under- or overestimated. Similarly, the GC content is also consistent with values inferred for other amphisbaenians (41-47%). The consistency in genome size with other squamate species supports the reliability of our assembly and suggests that *A. alba* does not deviate from the typical mitogenomic architecture observed in Squamata. 

The gene order in vertebrate mitogenomes is as conserved as their gene content; nevertheless, diverse types of rearrangements - such as gene transpositions, inversions, duplications, and losses - have been documented across a wide range of taxa (see Mindell et al., 1998; Boore, 1999; Boore and Brown, 1998; Zhong et al., 2005; Zhang et al., 2021). Here, we also analyzed the mitogenome of one amphisbaenian recently sequenced, *Blanus cinereus*, that was not included in the comparative studies aforementioned, and we did not identify any structural rearrangement in this species. Macey et al. (2004) investigated the evolution of mitochondrial DNA structural features among amphisbaenians and identified several remarkable genomic configurations. In contrast to most vertebrates, *Rhineura floridana* (family Rhineuridae) exhibits an inversion in which the *ND6-tRNA* ^*Glu*^ block shifted in order with the *CYTB-tRNA* ^*Thr*^ -tRNA^*Pro*^ block, a gene arrangement that closely resembles that found in birds (Mindell *et al*., 1998; Macey *et al*. 2004). In the Bipedidae, a derived mitochondrial gene organization has evolved through a shift involving *tRNA* ^*Glu*^ and *nad6*. Moreover, *Bipes biporus* shows a tandem duplication of *tRNA* ^*Thr*^ and *tRNA* ^*Pro*^ (Macey *et al*., 2004). Structural genomic variations reflect functional and evolutionary constraints and can arise from transcriptional or replication-related processes (Boore, 2000; Gissi et al., 2008). They may profoundly affect gene expression and replication, but the functional consequences of that merit further investigation (Gissi *et al*., 2008). 

Modifications in the evolutionary dynamics of mitochondrial proteins seem associated with lineages that likely experienced shifts in energy metabolism linked to changes in locomotor habits, as those derived from processes of limb reduction and loss (Wang et al., 2021; Wu et al. 2022), and to ecological transitions, including fossoriality (Silva et al., 2009; Tomasco and Lessa, 2011, 2014; Gan et al., 2018; Tavares and Seuánez, 2018). Our analyses corroborate the hypothesis that fossorial and limbless squamate lineages experienced particular selective regimes acting on the evolution of mitochondrial protein-coding genes. However, while we identified heterogeneous patterns in limbless lineages when compared to their fully-limbed counterparts - ranging from relaxed selection to stronger selection, and also no detectable differences - the trends associated with fossorial lineages were remarkably more consistent, as analyses supported intensification of selective pressures for most genes in this group when compared to non-fossorial groups.

When all genes were considered together, we identified strong selectiveness in the mitogenomes of the fossorial group, followed by limbless-fossorial lineages. In contrast, the analyses suggested relaxed selection in limbless squamate lineages relative to the background. Adaptation to fossoriality may be associated with intensified purifying selection on mitochondrial function, possibly reflecting the increased energetic demands of a burrowing lifestyle (Abe and Johansen, 1987). Depending on how deep the animals burrow, it is possible that they face reduced oxygen percentages in the underground environment (Lacey, 2000; Vihar et al., 2015); the intensified purifying selection in the mitogenomes of these species may relate to increased tolerance to hypoxia (Begall et al., 2007; Luo et al., 2008; Davies et al., 2015; Tavares and Seuánez, 2018) and enhanced capacities for sustained muscular activity underground (Klein and Codd, 2010). Besides, subterranean habitats impose other physiological challenges, such hypercapnia [high carbon dioxide - CO2], high humidity, and limited or no exposure to sunlight (Nevo, 1979; de Vries et al., 2008; Davies *et al*. 2015). *Amphisbaena alba* exhibits high concentrations of myoglobin in skeletal muscles and the heart (Weber et al., 1981), and the process of modifying the mitochondrial inner compartment limiting membrane into a lamellated body during the initial stages of the erythroid cell maturation seems to be slower in this amphisbaenian when compared to snakes (Spadacci-Morena et al., 1998). Although scarce, the existing information about energetic relationships in amphisbaenians supports functional interpretations of selective pressures associated with fossoriality during the evolution of mitochondrial genomes in Squamata.

The reduction or complete loss of limbs also may impose additional energetic costs to locomotion, regardless of fossoriality or not (Escalante et al., 2021; Wang et al., 2021; Wu et al., 2022). Previous studies reported accelerated evolution in *ATP6* among limbless lineages in Squamata (Wang *et al*., 2021), and positive selection has also been detected in the gene *ND2* of limbless skinks, when compared to limbed relatives (Wu *et al*., 2022). However, our results suggest that signals of positive selection are stronger in fossorial lineages, when compared to limbless species, given the larger number of genes identified as evolving under non-neutral regimes in fossorial squamates. This finding might relate to the great energetic demands of burrowing (i.e., fossoriality), which may overlap those involved in locomotion over the surface by limbless lineages. Moreover, while we identified signals of relaxed purifying selection in several loci of the mitogenome in limbless squamates, in fossorial lineages our results provide widespread evidence of intensified selection, supporting the idea that burrowing imposes consistent energetic challenges that shape the evolutionary trajectory of mitochondrial genes. Signal of intensified selection was particularly evident in limbless-fossorial species, with most protein-coding genes supporting stronger selection relative to the background. This pattern aligns with previous studies showing that the hypoxic and energetically demanding subterranean environments can drive adaptive shifts in mitochondrial function across diverse vertebrate groups (e.g., Silva et al., 2009; Tomasco and Lessa, 2011, 2014; Gan et al., 2018; Tavares and Seuánez, 2018). Our study suggests that the combination of ecological and morphological specialization in limbless-fossorial lineages may drive the strongest signal of selection in the mitogenomes of Squamata. Overall, our findings suggest that fossoriality exerts a more decisive influence than limb loss to establish the selective regimes acting during the evolution of mitochondrial genomes in Squamata, with potential implications for understanding how interactions among ecological pressures and morphological specializations shape the molecular evolution of squamates. Future studies integrating physiological performance, nuclear-mitochondrial interactions, and population-level data may further elucidate the mechanisms integrating ecology and mitochondrial genome evolution. 

By including a newly sequenced mitochondrial genome of *Amphisbaena alba* into comparative analyses across Squamata, we identified a strong and more pervasive influence of fossoriality in the evolution of mitochondrial genes, which surpasses the impact of limblessness alone. Our findings highlight the importance of ecological settings shaping selective regimes during mitogenome evolution, and suggest that lifestyle-driven energetic constraints may play a key role during the repeated evolution of extreme body plans in vertebrates.

## Supplementary material

The following online material is available for this article:

Figure S1 - Gene order and synteny of mitochondrial genomes in representative amphisbaenian species and the limbed outgroup (*Lacerta agilis*), belonging to Lacertidae, the sister lineage to Amphisbaenia.

Figure S2 - Mitochondrial genome size across squamates

Figure S3 - Site-specific selection patterns across mitochondrial proteins in squamate species.

Figure S4 - Results of RELAX analyses.

Table S1 - Database, including sampled species and associated information for genome size, presence in the phylogeny proposed by Title et al. (2024), inclusion or not in selection analyses, identification for the genome sequence accessed, classification concerning fossoriality and limblessness, family and squamate group.

Table S2 - Results for proportion of invariant sites and substitution saturation analyses of the selected squamate species assessed using DAMBE.

Table S3 - Selection analyses of mitochondrial genes in selected squamate species based on PAML.

Table S4 - Selection analyses of mitochondrial genes in selected squamate species based on PAML and ETE3 lineage-specific ω estimates.

Table S5 - Selection analyses of mitochondrial genes were performed using branch-site models in selected squamate lineages, with lineage-specific ω estimates obtained using PAML and ETE3.

Table S6 - Results of HyPhy’s RELAX.

## Data Availability

 Sequences of Amphisbaena alba mitogenomes obtained in our laboratory were deposited in GenBank (accession number PZ210514). Data provided also as electronic supplementary material.
