# Supplementary material for: Evolutionary patterns in squamate mitogenomes: Are selective regimes associated with fossoriality and limblessness?
Source: Genet Mol Biol. 2026 Jul 20;49(Suppl 2):e20250226. doi: 10.1590/1678-4685-GMB-2025-0226 (PMC13384248; doi:10.1590/1678-4685-GMB-2025-0226)
Supplement: Figure S1 - [file 1415-4757-GMB-49-s2-e20250226-s1.pdf]

Genetics and Molecular Biology
https://doi.org/10.1590/1678-4685-GMB-2025-0226

Supplementary Material to "Evolutionary patterns in squamate mitogenomes: are selective regimes associated with fossoriality and limblessness?"

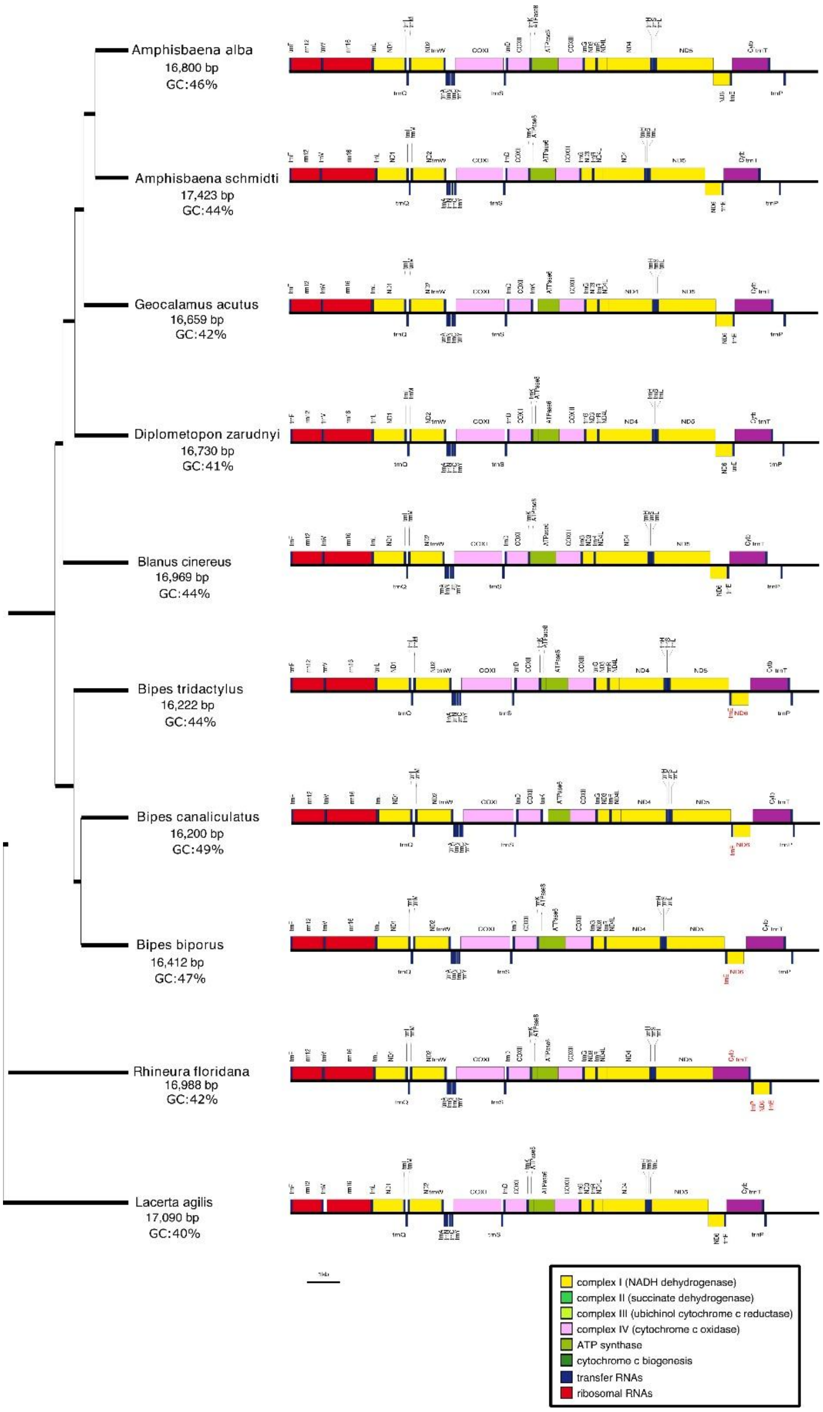

Figure S1 - Gene order and synteny of mitochondrial genomes in representative amphisbaenian species and the limbed outgroup (Lacerta agilis), belonging to Lacertidae, the sister lineage to Amphisbaenia. Protein-coding genes are shown as colored arrows indicating transcriptional direction (ND genes in green, COX genes in red, ATPase genes in blue, and CYTB in purple). Ribosomal RNA genes (rrnS and rrnL) are shown in brown, and transfer RNA genes are depicted as gray arrows. Gene order conservation among genomes is indicated by gray connecting lines. The phylogenetic relationships among species are shown on the left, and the scale bar represents 2 kb.
