# Supplementary material for: Evolutionary patterns in squamate mitogenomes: Are selective regimes associated with fossoriality and limblessness?
Source: Genet Mol Biol. 2026 Jul 20;49(Suppl 2):e20250226. doi: 10.1590/1678-4685-GMB-2025-0226 (PMC13384248; doi:10.1590/1678-4685-GMB-2025-0226)
Supplement: Figure S2 - [file 1415-4757-GMB-49-s2-e20250226-s2.pdf]

Genetics and Molecular Biology
https://doi.org/10.1590/1678-4685-GMB-2025-0226

Supplementary Material to "Evolutionary patterns in squamate mitogenomes: are selective regimes associated with fossoriality and limblessness?"

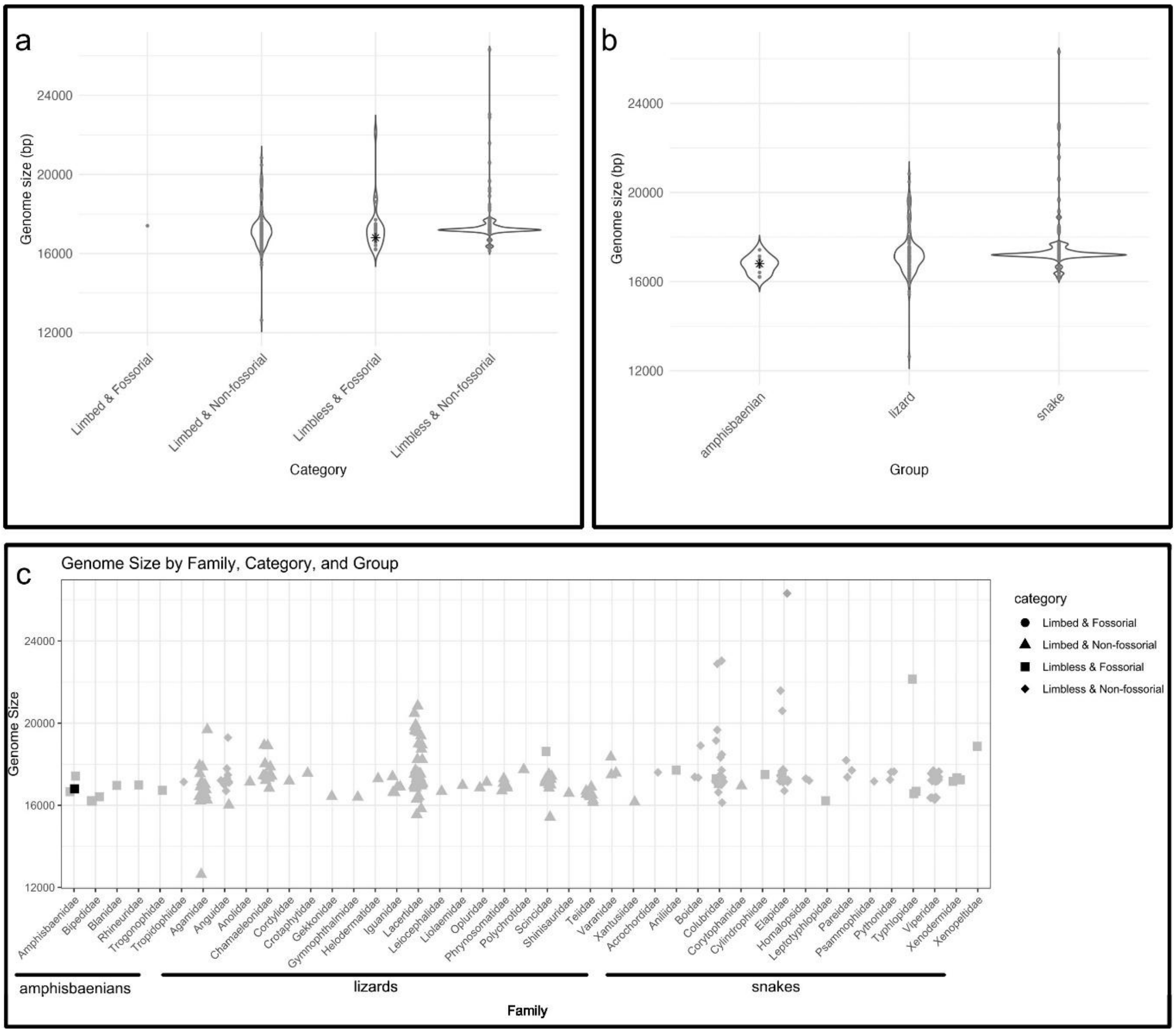

Figure S2 – Mitochondrial genome size across squamates. (a) Genome size by ecological category (limbed fossorial, limbed non-fossorial, limbless fossorial, limbless non-fossorial). The asterisk marks Amphisbaena alba, which falls within the range of limbless-fossorial species. (b) Genome size across major squamate groups (Amphisbaenia, lizards, and snakes). Amphisbaena alba is highlighted with an asterisk and lies within the amphisbaenian range. The five largest genomes correspond to snakes: Hydrophis ornatus (26,316 bp), Leptodeira polysticta (23,038 bp), Sibon nebulatus (22,887 bp), Indotyphlops braminus (22,144 bp), and Sinomicrurus japonicus (22,144 bp). (c) Genome size grouped by family, with Amphisbaena alba indicated in black.
