# Supplementary material for: Evolutionary patterns in squamate mitogenomes: Are selective regimes associated with fossoriality and limblessness?
Source: Genet Mol Biol. 2026 Jul 20;49(Suppl 2):e20250226. doi: 10.1590/1678-4685-GMB-2025-0226 (PMC13384248; doi:10.1590/1678-4685-GMB-2025-0226)
Supplement: Figure S3 - [file 1415-4757-GMB-49-s2-e20250226-s3.pdf]

Genetics and Molecular Biology
https://doi.org/10.1590/1678-4685-GMB-2025-0226

Supplementary Material to "Evolutionary patterns in squamate mitogenomes: are selective regimes associated with fossoriality and limblessness?"

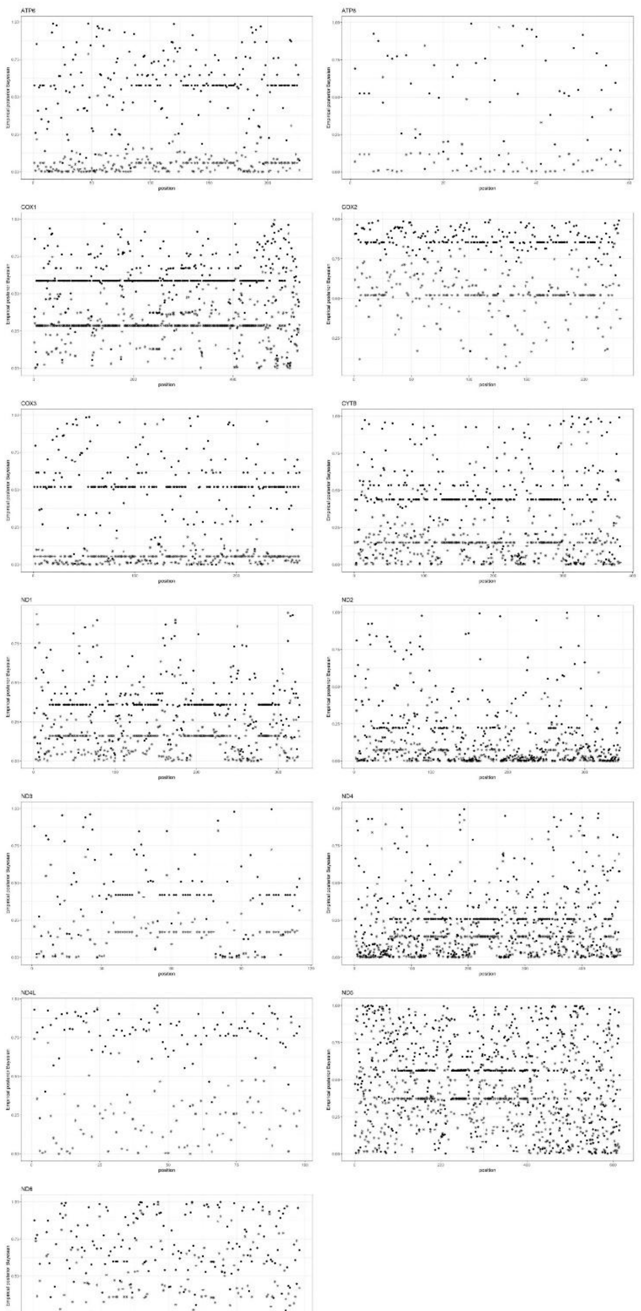

Figure S3 – Site-specific selection patterns across mitochondrial proteins in squamate species. For each gene (ATP6, ATP8, COX1–3, CYTB, ND1–6, and ND4L), the x-axis represents amino acid position and the y-axis shows the Empirical Bayes posterior probability of trait-associated shifts in evolutionary rate. Each point corresponds to an individual amino acid.
