# Supplementary material for: Evolutionary patterns in squamate mitogenomes: Are selective regimes associated with fossoriality and limblessness?
Source: Genet Mol Biol. 2026 Jul 20;49(Suppl 2):e20250226. doi: 10.1590/1678-4685-GMB-2025-0226 (PMC13384248; doi:10.1590/1678-4685-GMB-2025-0226)
Supplement: Figure S4 - [file 1415-4757-GMB-49-s2-e20250226-s4.pdf]

Genetics and Molecular Biology

https://doi.org/10.1590/1678-4685-GMB-2025-0226

Supplementary Material to "Evolutionary patterns in squamate mitogenomes: are selective regimes associated with fossoriality and limblessness?"

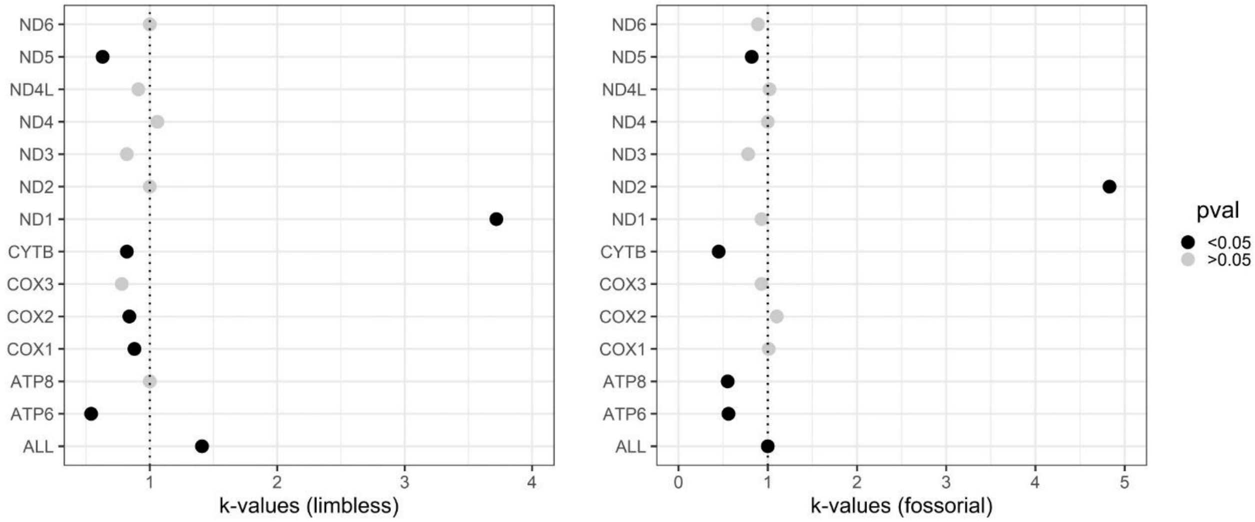

Figure S4 – Results of RELAX analyses. The x-axis shows the selection intensity parameter (k), where values of k > 1 indicate intensified selection and k < 1 indicate relaxed selection; each point represents a mitochondrial protein-coding gene analyzed across squamate lineages, separated according to limblessness (left) and fossoriality (right).
