# Supplementary material for: Evolutionary patterns in squamate mitogenomes: Are selective regimes associated with fossoriality and limblessness?
Source: Genet Mol Biol. 2026 Jul 20;49(Suppl 2):e20250226. doi: 10.1590/1678-4685-GMB-2025-0226 (PMC13384248; doi:10.1590/1678-4685-GMB-2025-0226)
Supplement: Table S1 - [file 1415-4757-GMB-49-s2-e20250226-s5.pdf]

Genetics and Molecular Biology
https://doi.org/10.1590/1678-4685-GMB-2025-0226

Supplementary Material to "Evolutionary patterns in squamate mitogenomes: are selective regimes associated with fossoriality and limblessness?"

Table S1 – Database, including sampled species and associated information for genome size, presence in the phylogeny proposed by Title et al. (2024), inclusion or not in selection analyses, identification for the genome sequence accessed, classification concerning fossoriality and limblessness, family and squamate group.

Species	Genome size	Phylogeny	Selection analyses	GenBank accession number	Fossoriality	Limblessness	Family	Group
Amphisbaena alba	16800	present	USED		Fully-fossorial	limbless	Amphisbaenidae	amphisbaenian
Amphisbaena schmidti	17423	present	USED	NC_006284.1	Fully-fossorial	limbless	Amphisbaenidae	amphisbaenian
Bipes biporus	16412	present	USED	NC_006287.1	Fully-fossorial	reduced	Bipedidae	amphisbaenian
Bipes canaliculatus	16200	present	USED	NC_006288.1	Fully-fossorial	reduced	Bipedidae	amphisbaenian
Bipes tridactylus	16222	present	USED	NC_006286.1	Fully-fossorial	reduced	Bipedidae	amphisbaenian
Blanus cinereus	16969	present	USED	NC_012433.1	Fully-fossorial	limbless	Blanidae	amphisbaenian
Diplometopon zarudnyi	16730	present	USED	NC_006283.1	Fully-fossorial	limbless	Trogonophidae	amphisbaenian
Geocalamus acutus	16659	present	USED	NC_006285.1	Fully-fossorial	limbless	Amphisbaenidae	amphisbaenian
Rhineura floridana	16988	present	USED	NC_006282.1	Fully-fossorial	limbless	Rhineuridae	amphisbaenian
Tropidophis haetianus	17138	present	USED	NC_012573.1	Non-fossorial	limbless	Tropidophiidae	snake
Ablepharus himalayanus	17304	present	not used	NC_058309.1	Non-fossorial	limbed	Scincidae	lizard
Ablepharus sikimmensis		absent	not used	NC_087854.1	Non-fossorial	limbed	Scincidae	lizard
Abronia graminea	16016	present	USED	NC_005958.1	Non-fossorial	limbed	Anguidae	lizard
Acanthodactylus aureus	17021	present	not used	NC_059775.1	Non-fossorial	limbed	Lacertidae	lizard
Acanthodactylus boskianus	17143	present	USED	NC_059772.1	Non-fossorial	limbed	Lacertidae	lizard
Acanthodactylus erythrurus	16827	present	not used	NC_059773.1	Non-fossorial	limbed	Lacertidae	lizard
Acanthodactylus guineensis	16963	present	not used	NC_059781.1	Non-fossorial	limbed	Lacertidae	lizard
Acanthodactylus schmidti	16943	present	not used	NC_059782.1	Non-fossorial	limbed	Lacertidae	lizard
Algyroides nigropunctatus	15844	present	not used	NC_059780.1	Non-fossorial	limbed	Lacertidae	lizard
Amblyrhynchus cristatus	16897	present	not used	NC_028031.1	Non-fossorial	limbed	Iguanidae	lizard
Anguis cephallonica	17208	present	USED	NC_030273.1	Non-fossorial	limbless	Anguidae	lizard
Anguis colchica	17097	present	not used	NC_048976.1	Non-fossorial	limbless	Anguidae	lizard
Anguis fragilis	17479	present	not used	NC_012431.1	Non-fossorial	limbless	Anguidae	lizard
Anguis graeca	19297	present	USED	NC_048977.1	Non-fossorial	limbless	Anguidae	lizard
Anguis veronensis	17322	present	USED	NC_048956.1	Non-fossorial	limbless	Anguidae	lizard
Anolis punctatus	17132	present	not used	NC_044125.1	Non-fossorial	limbed	Anolidae	lizard
Aspidoscelis dixoni		absent	not used	NC_067813.1	Non-fossorial	limbed	Teiidae	lizard
Aspidoscelis exsanguis		absent	not used	NC_067817.1	Non-fossorial	limbed	Teiidae	lizard
Aspidoscelis gularis	16507	present	not used	NC_067815.1	Non-fossorial	limbed	Teiidae	lizard
Aspidoscelis inornatus	16419	present	not used	NC_067819.1	Non-fossorial	limbed	Teiidae	lizard
Aspidoscelis laredoensis	16141	present	not used	NC_067823.1	Non-fossorial	limbed	Teiidae	lizard
Aspidoscelis marmoratus	16543	present	not used	NC_067820.1	Non-fossorial	limbed	Teiidae	lizard

Species	Genome size	Phylogeny	Selection analyses	GenBank accession number	Fossoriality	Limblessness	Family	Group
Aspidoscelis neomexicanus		absent	not used	NC_067818.1	Non-fossorial	limbed	Teiidae	lizard
Aspidoscelis neotesselatus	16542	present	not used	NC_067814.1	Non-fossorial	limbed	Teiidae	lizard
Aspidoscelis scalaris	16252	present	not used	NC_067821.1	Non-fossorial	limbed	Teiidae	lizard
Aspidoscelis sexlineatus	16893	present	not used	NC_067824.1	Non-fossorial	limbed	Teiidae	lizard
Aspidoscelis tesselatus	16693	present	not used	NC_067816.1	Non-fossorial	limbed	Teiidae	lizard
Aspidoscelis velox	16493	present	not used	NC_067822.1	Non-fossorial	limbed	Teiidae	lizard
Ateuchosaurus chinensis	16840	present	not used	NC_057221.1	Non-fossorial	limbed	Scincidae	lizard
Australolacerta australis	17019	present	not used	NC_059777.1	Non-fossorial	limbed	Lacertidae	lizard
Brookesia decaryi	17324	present	not used	NC_014174.1	Non-fossorial	limbed	Chamaeleonidae	lizard
Calotes mystaceus	16506	present	not used	NC_053755.1	Non-fossorial	limbed	Agamidae	lizard
Calotes versicolor	16670	present	not used	NC_009683.1	Non-fossorial	limbed	Agamidae	lizard
Chalarodon madagascariensis	16851	present	not used	NC_012836.1	Non-fossorial	limbed	Opluridae	lizard
Chamaeleo africanus	17415	present	not used	NC_012422.1	Non-fossorial	limbed	Chamaeleonidae	lizard
Chamaeleo arabicus	17400	present	not used	NC_012445.1	Non-fossorial	limbed	Chamaeleonidae	lizard
Chamaeleo calcaricarens	17451	present	not used	NC_012452.1	Non-fossorial	limbed	Chamaeleonidae	lizard
Chamaeleo calyptratus	17433	present	not used	NC_012420.1	Non-fossorial	limbed	Chamaeleonidae	lizard
Chamaeleo chamaeleon	17470	present	not used	NC_012427.1	Non-fossorial	limbed	Chamaeleonidae	lizard
Chamaeleo dilepis	17875	present	not used	NC_012436.1	Non-fossorial	limbed	Chamaeleonidae	lizard
Chamaeleo monachus	18900	present	not used	NC_012443.1	Non-fossorial	limbed	Chamaeleonidae	lizard
Chamaeleo zeylanicus	18923	present	not used	NC_012444.1	Non-fossorial	limbed	Chamaeleonidae	lizard
Chlamydosaurus kingii	16761	present	not used	NC_009421.1	Non-fossorial	limbed	Agamidae	lizard
Conolophus subcristatus	16892	present	not used	NC_028030.1	Non-fossorial	limbed	Iguanidae	lizard
Cyclura pinguis	16631	present	not used	NC_027089.1	Non-fossorial	limbed	Iguanidae	lizard
Darevskia armeniaca	17521	present	not used	NC_046006.1	Non-fossorial	limbed	Lacertidae	lizard
Darevskia brauneri	16976	present	not used	NC_050005.1	Non-fossorial	limbed	Lacertidae	lizard
Darevskia caucasica	16343	present	not used	NC_049999.1	Non-fossorial	limbed	Lacertidae	lizard
Darevskia chlorogaster	17479	present	not used	NC_050004.1	Non-fossorial	limbed	Lacertidae	lizard
Darevskia clarkorum	16301	present	not used	NC_050002.1	Non-fossorial	limbed	Lacertidae	lizard
Darevskia daghestanica	17189	present	not used	NC_050003.1	Non-fossorial	limbed	Lacertidae	lizard
Darevskia dahli	17528	present	USED	NC_046007.1	Non-fossorial	limbed	Lacertidae	lizard
Darevskia derjugini	16960	present	not used	NC_049998.1	Non-fossorial	limbed	Lacertidae	lizard
Darevskia mixta	17532	present	not used	NC_046008.1	Non-fossorial	limbed	Lacertidae	lizard
Darevskia parvula	17510	present	not used	NC_046009.1	Non-fossorial	limbed	Lacertidae	lizard
Darevskia portschinskii	17529	present	not used	NC_046010.1	Non-fossorial	limbed	Lacertidae	lizard
Darevskia praticola	16418	present	not used	NC_050000.1	Non-fossorial	limbed	Lacertidae	lizard
Darevskia raddei	20478	present	not used	NC_050001.1	Non-fossorial	limbed	Lacertidae	lizard
Darevskia rudis	17534	present	not used	NC_046011.1	Non-fossorial	limbed	Lacertidae	lizard
Darevskia saxicola	17524	present	not used	NC_046012.1	Non-fossorial	limbed	Lacertidae	lizard
Darevskia valentini	17393	present	not used	NC_045934.1	Non-fossorial	limbed	Lacertidae	lizard
Diploderma flaviceps	17140	present	not used	NC_039541.1	Non-fossorial	limbed	Agamidae	lizard
Diploderma micangshanense		absent	not used	NC_056342.1	Non-fossorial	limbed	Agamidae	lizard
Dopasia gracilis	17788	present	USED	NC_030369.1	Non-fossorial	limbless	Anguidae	lizard
Dopasia hainanensis	17000	present	not used	NC_057268.1	Non-fossorial	limbless	Anguidae	lizard
Dopasia harti	17163	present	USED	NC_022700.1	Non-fossorial	limbless	Anguidae	lizard
Dopasia sokolovi	17137	present	USED	NC_071253.1	Non-fossorial	limbless	Anguidae	lizard
Eremias brenchleyi	19542	present	not used	NC_011764.1	Non-fossorial	limbed	Lacertidae	lizard

Species	Genome size	Phylogeny	Selection analyses	GenBank accession number	Fossoriality	Limblessness	Family	Group
Eremias dzungarica		absent	not used	NC_056302.1	Non-fossorial	limbed	Lacertidae	lizard
Eremias multiocellata	19385	present	not used	NC_025304.1	Non-fossorial	limbed	Lacertidae	lizard
Eremias nikolskii	20840	present	not used	NC_060561.1	Non-fossorial	limbed	Lacertidae	lizard
Eremias przewalskii	18225	present	USED	NC_025929.1	Non-fossorial	limbed	Lacertidae	lizard
Eremias scripta	19824	present	not used	NC_064329.1	Non-fossorial	limbed	Lacertidae	lizard
Eremias stummeri	19602	present	not used	NC_029878.1	Non-fossorial	limbed	Lacertidae	lizard
Eremias szczerbaki	19650	present	not used	NC_062143.1	Non-fossorial	limbed	Lacertidae	lizard
Eremias vermiculata	19914	present	not used	NC_025320.1	Non-fossorial	limbed	Lacertidae	lizard
Eremias yarkandensis	18743	present	not used	NC_060637.1	Non-fossorial	limbed	Lacertidae	lizard
Eutropis multifasciata	16994	present	not used	NC_063644.1	Non-fossorial	limbed	Scincidae	lizard
Furcifer oustaleti	18021	present	not used	NC_008777.1	Non-fossorial	limbed	Chamaeleonidae	lizard
Gallotia atlantica	15552	present	not used	NC_059771.1	Non-fossorial	limbed	Lacertidae	lizard
Gambelia wislizenii	17563	present	not used	NC_012831.1	Non-fossorial	limbed	Crotaphytidae	lizard
Gekko gecko	16435	present	not used	NC_007627.1	Non-fossorial	limbed	Gekkonidae	lizard
Heloderma suspectum	17296	present	USED	NC_008776.1	Non-fossorial	limbed	Helodermatidae	lizard
Holbrookia lacerata	16846	present	not used	NC_041001.1	Non-fossorial	limbed	Phrynosomatidae	lizard
Hydrosaurus amboinensis	19682	present	not used	NC_014178.1	Non-fossorial	limbed	Agamidae	lizard
Iguana delicatissima	17397	present	not used	NC_044899.1	Non-fossorial	limbed	Iguanidae	lizard
Iguana iguana	16615	present	not used	NC_002793.1	Non-fossorial	limbed	Iguanidae	lizard
Iphisa elegans	16397	present	not used	NC_048879.1	Non-fossorial	limbed	Gymnophthalmidae	lizard
Isopachys gyldenstolpei	18622	present	USED	NC_041147.1	Fully-fossorial	limbless	Scincidae	lizard
Kinyongia fischeri	17210	present	not used	NC_012465.1	Non-fossorial	limbed	Chamaeleonidae	lizard
Lacerta agilis	18997	present	USED	NC_021766.1	Non-fossorial	limbed	Lacertidae	lizard
Lacerta bilineata	17090	present	USED	NC_028440.1	Non-fossorial	limbed	Lacertidae	lizard
Lacerta viridis viridis	17086	present	USED	NC_008328.1	Non-fossorial	limbed	Lacertidae	lizard
Laudakia tuberculata	17015	present	not used	NC_087804.1	Non-fossorial	limbed	Agamidae	lizard
Leiocephalus personatus	16681	present	not used	NC_012834.1	Non-fossorial	limbed	Leiocephalidae	lizard
Leiolepis guttata	16552	present	not used	NC_014179.1	Non-fossorial	limbed	Agamidae	lizard
Leiolepis reevesii	16908	present	not used	NC_024179.1	Non-fossorial	limbed	Agamidae	lizard
Lepidophyma flavimaculatum	16158	present	not used	NC_008775.1	Non-fossorial	limbed	Xantusiidae	lizard
Liolaemus darwinii	16974	present	not used	NC_057242.1	Non-fossorial	limbed	Liolaemidae	lizard
Liolaemus millcayac		absent	not used	NC_057243.1	Non-fossorial	limbed	Liolaemidae	lizard
Liolaemus parthenos		absent	not used	NC_057244.1	Non-fossorial	limbed	Liolaemidae	lizard
Meroles squamulosus	16860	present	not used	NC_059779.1	Non-fossorial	limbed	Lacertidae	lizard
Mesalina olivieri	16899	present	not used	NC_059774.1	Non-fossorial	limbed	Lacertidae	lizard
Oplurus grandidieri	17122	present	not used	NC_012827.1	Non-fossorial	limbed	Opluridae	lizard
Pedioplanis laticeps	17046	present	not used	NC_059778.1	Non-fossorial	limbed	Lacertidae	lizard
Phoenicolacerta kulzeri	17199	present	not used	NC_011606.1	Non-fossorial	limbed	Lacertidae	lizard
Phrynocephalus albolineatus		absent	not used	NC_029186.1	Non-fossorial	limbed	Agamidae	lizard
Phrynocephalus axillaris	17937	present	not used	NC_020340.1	Non-fossorial	limbed	Agamidae	lizard
Phrynocephalus forsythii	17542	present	not used	NC_026454.1	Non-fossorial	limbed	Agamidae	lizard
Phrynocephalus grumgrzimailoi		absent	not used	NC_025640.1	Non-fossorial	limbed	Agamidae	lizard
Phrynocephalus guinanensis		absent	not used	NC_024875.1	Non-fossorial	limbed	Agamidae	lizard
Phrynocephalus helioscopus	16249	present	not used	NC_025639.1	Non-fossorial	limbed	Agamidae	lizard
Phrynocephalus helioscopus cameranoi		absent	not used	NC_061391.1	Non-fossorial	limbed	Agamidae	lizard
Phrynocephalus helioscopus varius		absent	not used	NC_061390.1	Non-fossorial	limbed	Agamidae	lizard

Species	Genome size	Phylogeny	Selection analyses	GenBank accession number	Fossoriality	Limblessness	Family	Group
Phrynocephalus maculatus	12631	present	not used	NC_057209.1	Non-fossorial	limbed	Agamidae	lizard
Phrynocephalus nasatus		absent	not used	NC_057064.1	Non-fossorial	limbed	Agamidae	lizard
Phrynocephalus przewalskii	16892	present	not used	NC_022719.1	Non-fossorial	limbed	Agamidae	lizard
Phrynocephalus putjatai	16283	present	not used	NC_024612.1	Non-fossorial	limbed	Agamidae	lizard
Phrynocephalus versicolor	16429	present	not used	NC_024654.1	Non-fossorial	limbed	Agamidae	lizard
Phrynosoma blainvillii	16946	present	not used	NC_036492.1	Non-fossorial	limbed	Phrynosomatidae	lizard
Plestiodon capito	17344	present	not used	NC_060292.1	Non-fossorial	limbed	Scincidae	lizard
Plestiodon chinensis	17175	present	not used	NC_029352.1	Non-fossorial	limbed	Scincidae	lizard
Plestiodon egregius	17407	present	USED	NC_000888.1	Semi-fossorial	limbed	Scincidae	lizard
Plestiodon elegans	17304	present	USED	NC_024576.1	Non-fossorial	limbed	Scincidae	lizard
Plestiodon liui		absent	not used	NC_070246.1	Non-fossorial	limbed	Scincidae	lizard
Plestiodon tunganus	17263	present	not used	NC_045232.1	Non-fossorial	limbed	Scincidae	lizard
Podarcis muralis	17311	present	USED	NC_011607.1	Non-fossorial	limbed	Lacertidae	lizard
Podarcis siculus	17297	present	USED	NC_011609.1	Non-fossorial	limbed	Lacertidae	lizard
Pogona vitticeps	16751	present	not used	NC_006922.1	Non-fossorial	limbed	Agamidae	lizard
Polychrus marmoratus	17743	present	not used	NC_012839.1	Non-fossorial	limbed	Polychrotidae	lizard
Psammodromus algirus	17118	present	not used	NC_059776.1	Non-fossorial	limbed	Lacertidae	lizard
Pseudocalotes microlepis	17873	present	not used	NC_039453.1	Non-fossorial	limbed	Agamidae	lizard
Pseudopus apodus	16701	present	USED	NC_071252.1	Non-fossorial	limbless	Anguidae	lizard
Pseudotrapelus sinaitus	16560	present	not used	NC_013603.2	Non-fossorial	limbed	Agamidae	lizard
Sceloporus occidentalis	17072	present	not used	NC_005960.1	Non-fossorial	limbed	Phrynosomatidae	lizard
Scincella huanrenensis	17212	present	not used	NC_030779.1	Non-fossorial	limbed	Scincidae	lizard
Scincella modesta	17511	present	not used	NC_048521.1	Non-fossorial	limbed	Scincidae	lizard
Scincella reevesii	15424	present	not used	NC_054206.1	Non-fossorial	limbed	Scincidae	lizard
Scincella vandenburghi	17103	present	not used	NC_030776.1	Non-fossorial	limbed	Scincidae	lizard
Shinisaurus crocodilurus	16583	present	USED	NC_005959.1	Non-fossorial	limbed	Shinisauridae	lizard
Smaug warreni	17184	present	not used	NC_005962.1	Non-fossorial	limbed	Cordylidae	lizard
Sphenomorphus incognitus	17417	present	USED	NC_041124.1	Non-fossorial	limbed	Scincidae	lizard
Sphenomorphus indicus	17027	present	not used	NC_045408.1	Non-fossorial	limbed	Scincidae	lizard
Takydromus amurensis	17333	present	not used	NC_030209.1	Non-fossorial	limbed	Lacertidae	lizard
Takydromus intermedius	17713	present	not used	NC_077637.1	Non-fossorial	limbed	Lacertidae	lizard
Takydromus sexlineatus	18943	present	USED	NC_022703.1	Non-fossorial	limbed	Lacertidae	lizard
Takydromus sylvaticus	17518	present	not used	NC_067055.1	Non-fossorial	limbed	Lacertidae	lizard
Takydromus wolteri	18236	present	not used	NC_018777.1	Non-fossorial	limbed	Lacertidae	lizard
Trioceros melleri	16832	present	not used	NC_014176.1	Non-fossorial	limbed	Chamaeleonidae	lizard
Tropidophorus hainanus	17001	present	not used	NC_066473.1	Non-fossorial	limbed	Scincidae	lizard
Tropidophorus hangnam		absent	not used	NC_050664.1	Non-fossorial	limbed	Scincidae	lizard
Uromastyx benti	16380	present	not used	NC_014182.1	Non-fossorial	limbed	Agamidae	lizard
Urosaurus nigricauda	17298	present	not used	NC_026308.1	Non-fossorial	limbed	Phrynosomatidae	lizard
Uta stansburiana	16703	present	not used	NC_027261.1	Non-fossorial	limbed	Phrynosomatidae	lizard
Varanus nebulosus	18347	present	USED	NC_073506.1	Non-fossorial	limbed	Varanidae	lizard
Varanus rudicollis	17575	present	USED	NC_066236.1	Non-fossorial	limbed	Varanidae	lizard
Varanus salvator	17489	present	USED	NC_010974.1	Non-fossorial	limbed	Varanidae	lizard
Xenagama taylori	16220	present	not used	NC_008065.1	Non-fossorial	limbed	Agamidae	lizard
Zootoca vivipara	17046	present	not used	NC_026867.1	Non-fossorial	limbed	Lacertidae	lizard
Achalinus meiguensis	17239	present	USED	NC_011576.1	Fully-fossorial	limbless	Xenodermidae	snake

Species	Genome size	Phylogeny	Selection analyses	GenBank accession number	Fossoriality	Limblessness	Family	Group
Achalinus rufescens	17339	present	USED	NC_032085.1	Fully-fossorial	limbless	Xenodermidae	snake
Achalinus spinalis	17165	present	USED	NC_032084.1	Fully-fossorial	limbless	Xenodermidae	snake
Acrochordus granulatus	17604	present	not used	NC_007400.1	Non-fossorial	limbless	Acrochordidae	snake
Agkistrodon contortrix	16269	present	not used	NC_035638.1	Non-fossorial	limbless	Viperidae	snake
Agkistrodon piscivorus	17213	present	not used	NC_009768.1	Non-fossorial	limbless	Viperidae	snake
Aipysurus eydouxii	17228	present	not used	NC_062614.1	Non-fossorial	limbless	Elapidae	snake
Amerotyphlops reticulatus	16681	present	USED	NC_010971.1	Fully-fossorial	limbless	Typhlopidae	snake
Anilius scytale	17713	present	USED	NC_014343.1	Fully-fossorial	limbless	Aniliidae	snake
Azemiops feae	17383	present	not used	NC_030781.1	Non-fossorial	limbless	Viperidae	snake
Basiliscus vittatus	16948	present	not used	NC_012829.1	Non-fossorial	limbed	Corytophanidae	snake
Boa constrictor	18905	present	not used	NC_007398.1	Non-fossorial	limbless	Boidae	snake
Boiga kraepelini	17124	present	not used	NC_070009.1	Non-fossorial	limbless	Colubridae	snake
Bothrops diporus	17642	present	not used	NC_039649.1	Non-fossorial	limbless	Viperidae	snake
Bothrops jararaca	17526	present	not used	NC_030760.1	Non-fossorial	limbless	Viperidae	snake
Bothrops pubescens	17694	present	not used	NC_039648.1	Non-fossorial	limbless	Viperidae	snake
Bungarus fasciatus	17234	present	not used	NC_011393.1	Non-fossorial	limbless	Elapidae	snake
Bungarus multicinctus	17139	present	not used	NC_084338.1	Non-fossorial	limbless	Elapidae	snake
Calamaria septentrionalis	17053	present	USED	NC_062677.1	Fully-fossorial	limbless	Colubridae	snake
Causus defilippii	17342	present	not used	NC_013479.1	Non-fossorial	limbless	Viperidae	snake
Chilabothrus argentum	17345	present	not used	NC_063114.1	Non-fossorial	limbless	Boidae	snake
Coluber constrictor	17142	present	not used	NC_071936.1	Non-fossorial	limbless	Colubridae	snake
Crotalus adamanteus	17242	present	not used	NC_041524.1	Non-fossorial	limbless	Viperidae	snake
Cylindrophis ruffus	17499	present	USED	NC_007401.1	Fully-fossorial	limbless	Cylindrophiidae	snake
Daboia russelii	17246	present	not used	NC_011391.1	Non-fossorial	limbless	Viperidae	snake
Deinagkistrodon acutus	17548	present	not used	NC_010223.1	Non-fossorial	limbless	Viperidae	snake
Dryophiops rubescens	17194	present	not used	NC_068689.1	Non-fossorial	limbless	Colubridae	snake
Echis carinatus	16365	present	not used	NC_060591.1	Non-fossorial	limbless	Viperidae	snake
Echis coloratus	16389	present	not used	NC_060592.1	Non-fossorial	limbless	Viperidae	snake
Echis omanensis	16372	present	not used	NC_063589.1	Non-fossorial	limbless	Viperidae	snake
Elaphe anomala	17164	present	not used	NC_027001.1	Non-fossorial	limbless	Colubridae	snake
Elaphe bimaculata	17183	present	not used	NC_024743.1	Non-fossorial	limbless	Colubridae	snake
Elaphe davidi	17117	present	not used	NC_025643.1	Non-fossorial	limbless	Colubridae	snake
Elaphe dione	17172	present	not used	NC_041068.1	Non-fossorial	limbless	Colubridae	snake
Elaphe druzei		absent	not used	NC_073568.1	Non-fossorial	limbless	Colubridae	snake
Elaphe moellendorffi	17191	present	not used	NC_063502.1	Non-fossorial	limbless	Colubridae	snake
Elaphe schrenckii	17165	present	not used	NC_027605.1	Non-fossorial	limbless	Colubridae	snake
Elaphe taeniura	17183	present	not used	NC_025275.1	Non-fossorial	limbless	Colubridae	snake
Elaphe urartica		absent	not used	NC_073569.1	Non-fossorial	limbless	Colubridae	snake
Emydocephalus ijimae	20598	present	not used	NC_066234.1	Non-fossorial	limbless	Elapidae	snake
Epicrates crassus	17379	present	not used	NC_083268.1	Non-fossorial	limbless	Boidae	snake
Euprepiophis perlaceus	17160	present	not used	NC_024546.1	Non-fossorial	limbless	Colubridae	snake
Fowlea piscator	16999	present	not used	NC_061951.1	Non-fossorial	limbless	Colubridae	snake
Gloydius brevicauda	17227	present	not used	NC_011390.1	Non-fossorial	limbless	Viperidae	snake
Gloydius himalayanus		absent	not used	NC_068353.1	Non-fossorial	limbless	Viperidae	snake
Gloydius rubromaculatus		absent	not used	NC_064056.1	Non-fossorial	limbless	Viperidae	snake
Gloydius shedaoensis	17218	present	not used	NC_029424.1	Non-fossorial	limbless	Viperidae	snake

Species	Genome size	Phylogeny	Selection analyses	GenBank accession number	Fossoriality	Limblessness	Family	Group
Gloydius strauchi	17222	present	not used	NC_036234.1	Non-fossorial	limbless	Viperidae	snake
Gloydius ussuriensis	17224	present	not used	NC_026553.1	Non-fossorial	limbless	Viperidae	snake
Gonyosoma frenatum	17208	present	not used	NC_057467.1	Non-fossorial	limbless	Colubridae	snake
Hebius craspedogaster	17209	present	not used	NC_070008.1	Non-fossorial	limbless	Colubridae	snake
Hebius metusia	17120	present	not used	NC_072535.1	Non-fossorial	limbless	Colubridae	snake
Hydrophis curtus	16705	present	not used	NC_046794.1	Non-fossorial	limbless	Elapidae	snake
Hydrophis cyanocinctus	17702	present	not used	NC_046795.1	Non-fossorial	limbless	Elapidae	snake
Hydrophis melanocephalus	17750	present	not used	NC_066232.1	Non-fossorial	limbless	Elapidae	snake
Hydrophis ornatus	26316	present	not used	NC_066233.1	Non-fossorial	limbless	Elapidae	snake
Hypsiglena chlorophaea chlorophaea		absent	not used	NC_013977.1	Non-fossorial	limbless	Colubridae	snake
Hypsiglena chlorophaea deserticola		absent	not used	NC_013989.1	Non-fossorial	limbless	Colubridae	snake
Hypsiglena jani texana		absent	not used	NC_013975.1	Non-fossorial	limbless	Colubridae	snake
Hypsiglena ochrorhyncha klauberi		absent	not used	NC_013984.1	Non-fossorial	limbless	Colubridae	snake
Hypsiglena ochrorhyncha nuchalata		absent	not used	NC_013983.1	Non-fossorial	limbless	Colubridae	snake
Hypsiglena ochrorhyncha ochrorhyncha		absent	not used	NC_013980.1	Non-fossorial	limbless	Colubridae	snake
Hypsiglena slevini	16129	present	not used	NC_013987.1	Non-fossorial	limbless	Colubridae	snake
Hypsiglena sp. DGM-2008		absent	not used	NC_013982.1	Non-fossorial	limbless	Colubridae	snake
Hypsiglena torquata	17197	present	not used	NC_013992.1	Non-fossorial	limbless	Colubridae	snake
Hypsiglena unaocularus		absent	not used	NC_024164.1	Non-fossorial	limbless	Colubridae	snake
Hypsiscopus plumbea	17203	present	not used	NC_010200.1	Non-fossorial	limbless	Homalopsidae	snake
Imantodes cenchoa	16633	present	not used	NC_013988.1	Non-fossorial	limbless	Colubridae	snake
Indotyphlops braminus	22144	present	USED	NC_010196.1	Fully-fossorial	limbless	Typhlopidae	snake
Lachesis muta	17177	present	not used	NC_081003.1	Non-fossorial	limbless	Viperidae	snake
Laticauda colubrina	17450	present	not used	NC_036054.1	Non-fossorial	limbless	Elapidae	snake
Laticauda laticaudata	17209	present	not used	NC_036053.1	Non-fossorial	limbless	Elapidae	snake
Laticauda semifasciata	17170	present	not used	NC_036055.1	Non-fossorial	limbless	Elapidae	snake
Leptodeira polysticta	23038	present	not used	NC_013990.1	Non-fossorial	limbless	Colubridae	snake
Lycodon flavozonatus	17172	present	not used	NC_028730.1	Non-fossorial	limbless	Colubridae	snake
Lycodon rufozonatus	17188	present	not used	NC_024559.1	Non-fossorial	limbless	Colubridae	snake
Lycodon ruhstrati	17168	present	not used	NC_046046.1	Non-fossorial	limbless	Colubridae	snake
Lycodon semicarinatus	17191	present	not used	NC_001945.1	Non-fossorial	limbless	Colubridae	snake
Macrovipera schweizeri	17152	present	not used	NC_044966.1	Non-fossorial	limbless	Viperidae	snake
Malayopython reticulatus	17641	present	USED	NC_042397.1	Non-fossorial	limbless	Pythonidae	snake
Micrurus fulvius	17506	present	USED	NC_013481.1	Fully-fossorial	limbless	Elapidae	snake
Myanophis thanlyinensis		absent	not used	NC_060375.1	Non-fossorial	limbless	Homalopsidae	snake
Myrrophis chinensis	17302	present	not used	NC_072633.1	Non-fossorial	limbless	Homalopsidae	snake
Naja atra	17216	present	not used	NC_011389.1	Non-fossorial	limbless	Elapidae	snake
Naja naja	17213	present	not used	NC_010225.1	Non-fossorial	limbless	Elapidae	snake
Nerodia sipedon	17706	present	not used	NC_015793.1	Non-fossorial	limbless	Colubridae	snake
Oligodon chinensis	17146	present	USED	NC_052835.1	Non-fossorial	limbless	Colubridae	snake
Oocatochus rufodorsatus	17159	present	not used	NC_022146.1	Non-fossorial	limbless	Colubridae	snake
Ophiophagus hannah	17267	present	not used	NC_011394.1	Non-fossorial	limbless	Elapidae	snake
Opisthotropis guangxiensis	17042	present	not used	NC_056274.1	Non-fossorial	limbless	Colubridae	snake
Opisthotropis latouchii	17051	present	not used	NC_046823.1	Non-fossorial	limbless	Colubridae	snake
Oreocryptophis porphyraceus	17167	present	not used	NC_012770.1	Non-fossorial	limbless	Colubridae	snake
Orientocoluber spinalis	17196	present	not used	NC_049067.1	Non-fossorial	limbless	Colubridae	snake

Species	Genome size	Phylogeny	Selection analyses	GenBank accession number	Fossoriality	Limblessness	Family	Group
Ovophis okinavensis	17388	present	not used	NC_007397.1	Non-fossorial	limbless	Viperidae	snake
Pantherophis slowinskii	17189	present	not used	NC_009769.1	Non-fossorial	limbless	Colubridae	snake
Pareas boulengeri	18190	present	USED	NC_050894.1	Non-fossorial	limbless	Pareidae	snake
Pareas formosensis	17703	present	USED	NC_058002.1	Non-fossorial	limbless	Pareidae	snake
Pareas stanleyi	17372	present	USED	NC_057676.1	Non-fossorial	limbless	Pareidae	snake
Plagiopholis styani	19669	present	not used	NC_060420.1	Non-fossorial	limbless	Colubridae	snake
Protobothrops cornutus	17219	present	not used	NC_022695.1	Non-fossorial	limbless	Viperidae	snake
Protobothrops dabieshanensis	17193	present	not used	NC_022473.1	Non-fossorial	limbless	Viperidae	snake
Protobothrops flavoviridis	17232	present	not used	NC_030181.1	Non-fossorial	limbless	Viperidae	snake
Protobothrops himalayanus	17389	present	not used	NC_029165.1	Non-fossorial	limbless	Viperidae	snake
Protobothrops jerdonii	17239	present	not used	NC_021402.1	Non-fossorial	limbless	Viperidae	snake
Protobothrops kaulbacki	17237	present	not used	NC_029166.1	Non-fossorial	limbless	Viperidae	snake
Protobothrops mangshanensis	17230	present	not used	NC_026052.1	Non-fossorial	limbless	Viperidae	snake
Protobothrops maolanensis	17228	present	not used	NC_026051.1	Non-fossorial	limbless	Viperidae	snake
Protobothrops mucrosquamatus	17234	present	not used	NC_021412.1	Non-fossorial	limbless	Viperidae	snake
Protobothrops tokarensis	17233	present	not used	NC_030182.1	Non-fossorial	limbless	Viperidae	snake
Psammophis lineolatus	17166	present	not used	NC_061027.1	Non-fossorial	limbless	Psammophiidae	snake
Pseudagkistrodon rudis	19153	present	not used	NC_056928.1	Non-fossorial	limbless	Colubridae	snake
Pseudoleptodeira latifasciata	18329	present	not used	NC_013981.1	Non-fossorial	limbless	Colubridae	snake
Pseudoxenodon stejnegeri	18475	present	not used	NC_053908.1	Non-fossorial	limbless	Colubridae	snake
Ptyas dhumnades	17164	present	not used	NC_028049.1	Non-fossorial	limbless	Colubridae	snake
Ptyas korros	17169	present	not used	NC_070409.1	Non-fossorial	limbless	Colubridae	snake
Ptyas major	17217	present	not used	NC_028048.1	Non-fossorial	limbless	Colubridae	snake
Ptyas mucosa	17151	present	not used	NC_030041.1	Non-fossorial	limbless	Colubridae	snake
Python bivittatus	17617	present	USED	NC_021479.1	Non-fossorial	limbless	Pythonidae	snake
Python molurus molurus		absent	not used	NC_015812.1	Non-fossorial	limbless	Pythonidae	snake
Python regius	17245	present	not used	NC_007399.1	Non-fossorial	limbless	Pythonidae	snake
Rena humilis	16218	present	USED	NC_005961.1	Fully-fossorial	limbless	Leptotyphlopidae	snake
Rhabdophis tigrinus	17415	present	not used	NC_030210.1	Non-fossorial	limbless	Colubridae	snake
Sibon nebulatus	22887	present	not used	NC_013985.1	Non-fossorial	limbless	Colubridae	snake
Sibynophis chinensis	17163	present	not used	NC_022430.1	Non-fossorial	limbless	Colubridae	snake
Sibynophis collaris	17163	present	not used	NC_016424.1	Non-fossorial	limbless	Colubridae	snake
Sinomicrurus japonicus	21581	present	USED	NC_066235.1	Non-fossorial	limbless	Elapidae	snake
Sinomicrurus macclellandi	17120	present	not used	NC_054255.1	Non-fossorial	limbless	Elapidae	snake
Sistrurus catenatus	17245	present	not used	NC_071935.1	Non-fossorial	limbless	Viperidae	snake
Stichophanes ningshaanensis	17292	present	not used	NC_026083.1	Semi-fossorial	limbless	Colubridae	snake
Thermophis baileyi	17355	present	not used	NC_035713.1	Non-fossorial	limbless	Colubridae	snake
Thermophis shangrila		absent	not used	NC_035058.1	Non-fossorial	limbless	Colubridae	snake
Thermophis zhaoermii	17322	present	not used	NC_012816.1	Non-fossorial	limbless	Colubridae	snake
Trimeresurus albolabris	17220	present	not used	NC_022820.1	Non-fossorial	limbless	Viperidae	snake
Trimeresurus sichuanensis	17225	present	not used	NC_029494.1	Non-fossorial	limbless	Viperidae	snake
Trimeresurus stejnegeri stejnegeri		absent	not used	NC_012146.1	Non-fossorial	limbless	Viperidae	snake
Trimerodytes annularis		absent	not used	NC_063962.1	Non-fossorial	limbless	Colubridae	snake
Vipera berus	16370	present	not used	NC_036956.1	Non-fossorial	limbless	Viperidae	snake
Xenopeltis unicolor	18872	present	USED	NC_007402.1	Semi-fossorial	limbless	Xenopeltidae	snake
Xerotyphlops vermicularis	16568	present	USED	NC_044967.1	Fully-fossorial	limbless	Typhlopidae	snake
