# Supplementary material for: Evolutionary patterns in squamate mitogenomes: Are selective regimes associated with fossoriality and limblessness?
Source: Genet Mol Biol. 2026 Jul 20;49(Suppl 2):e20250226. doi: 10.1590/1678-4685-GMB-2025-0226 (PMC13384248; doi:10.1590/1678-4685-GMB-2025-0226)
Supplement: Table S2 - [file 1415-4757-GMB-49-s2-e20250226-s6.pdf]

Genetics and Molecular Biology
https://doi.org/10.1590/1678-4685-GMB-2025-0226

Supplementary Material to "Evolutionary patterns in squamate mitogenomes: are selective regimes associated with fossoriality and limblessness?"

Table S2 – Results for proportion of invariant sites and substitution saturation analyses of the selected squamate species assessed using DAMBE. Abbreviations: IS (%) = proportion of invariant sites; NumOTU = number of operational taxonomic units (taxa) included in the analysis; iss (index of substitution saturation) = observed index measuring the degree of substitution saturation in the alignment; iss.cSym = critical iss value expected under a symmetrical tree topology; T = student's *t* statistics testing whether the observed Iss is significantly lower than the corresponding Iss.c value; DF = Degrees of freedom; P = p-value; Iss.cAsym = critical Iss value expected under an asymmetrical (highly unbalanced) tree topology.

				Symmetrical topology model				Asymmetrical topology model			
	IS (%)	NumOTU	Iss	Iss.cSym	T	DF	P	Iss.cAsym	T	DF	P
ATP6	0.0089	4	0.514	0.806	12.744	674	<0.001	0.775	11.391	674	<0.001
		8	0.510	0.767	10.262	674	<0.001	0.658	5.896	674	<0.001
		16	0.518	0.747	8.687	674	<0.001	0.538	0.753	674	0.452
		32	0.524	0.721	7.209	674	<0.001	0.395	4.689	674	<0.001
ATP8	0.0010	4	0.759	0.778	0.396	173	0.693	0.777	0.368	173	0.713
		8	0.789	0.740	0.861	173	0.390	0.648	2.447	173	0.015
		16	0.822	0.631	2.740	173	0.007	0.453	5.276	173	<0.001
		32	0.860	0.701	1.895	173	0.060	0.398	5.522	173	<0.001
COX1	0.0291	4	0.382	0.835	29.202	1551	<0.001	0.806	27.303	1551	<0.001
		8	0.384	0.812	22.786	1551	<0.001	0.713	17.538	1551	<0.001
		16	0.396	0.795	18.439	1551	<0.001	0.615	10.104	1551	<0.001
		32	0.410	0.778	14.994	1551	<0.001	0.499	3.604	1551	<0.001
COX2	0.0124	4	0.450	0.807	15.961	683	<0.001	0.776	14.572	683	<0.001
		8	0.442	0.768	12.952	683	<0.001	0.659	8.616	683	<0.001
		16	0.444	0.748	11.083	683	<0.001	0.539	3.462	683	0.001
		32	0.453	0.722	9.065	683	<0.001	0.397	1.898	683	0.058
COX3	0.0163	4	0.370	0.812	21.708	772	<0.001	0.780	20.171	772	<0.001
		8	0.369	0.775	17.979	772	<0.001	0.667	13.191	772	<0.001
		16	0.373	0.757	15.843	772	<0.001	0.551	7.335	772	<0.001
		32	0.379	0.731	13.839	772	<0.001	0.412	1.273	772	0.203
CYTB	0.0191	4	0.449	0.825	21.352	1123	<0.001	0.793	19.540	1123	<0.001
		8	0.460	0.795	16.756	1123	<0.001	0.691	11.553	1123	<0.001
		16	0.463	0.779	14.509	1123	<0.001	0.586	5.633	1123	<0.001
		32	0.475	0.757	12.060	1123	<0.001	0.460	0.647	1123	0.518
ND1	0.0102	4	0.421	0.820	20.954	967	<0.001	0.788	19.273	967	<0.001
		8	0.429	0.787	16.242	967	<0.001	0.681	11.428	967	<0.001

				Symmetrical topology model				Asymmetrical topology model			
	IS (%)	NumOTU	Iss	Iss.cSym	T	DF	P	Iss.cAsym	T	DF	P
		16	0.436	0.770	13.713	967	<0.001	0.571	5.560	967	<0.001
		32	0.448	0.746	11.150	967	<0.001	0.439	0.315	967	0.753
ND2	0.0106	4	0.541	0.822	15.494	1026	<0.001	0.790	13.729	1026	<0.001
		8	0.544	0.790	12.660	1026	<0.001	0.685	7.252	1026	<0.001
		16	0.548	0.773	11.111	1026	<0.001	0.577	1.421	1026	0.155
		32	0.555	0.750	9.281	1026	<0.001	0.447	5.147	1026	<0.001
ND3	0.0780	4	0.529	0.784	7.811	317	<0.001	0.756	6.939	317	<0.001
		8	0.528	0.737	5.993	317	<0.001	0.627	2.842	317	0.005
		16	0.528	0.690	4.476	317	<0.001	0.482	1.284	317	0.200
		32	0.537	0.686	4.046	317	<0.001	0.356	4.923	317	<0.001
ND4	0.0109	4	0.519	0.831	18.965	1385	<0.001	0.801	17.088	1385	<0.001
		8	0.530	0.806	14.492	1385	<0.001	0.705	9.184	1385	<0.001
		16	0.542	0.789	11.512	1385	<0.001	0.603	2.835	1385	0.005
		32	0.557	0.770	8.955	1385	<0.001	0.484	3.081	1385	0.002
ND4L	0.0078	4	0.541	0.780	7.147	291	<0.001	0.756	6.423	291	<0.001
		8	0.548	0.734	5.260	291	<0.001	0.626	2.215	291	0.027
		16	0.552	0.676	3.432	291	0.001	0.471	2.220	291	0.027
		32	0.562	0.682	3.256	291	0.001	0.354	5.643	291	<0.001
ND5	0.0061	4	0.536	0.838	21.303	1836	<0.001	0.811	19.383	1836	<0.001
		8	0.547	0.819	16.833	1836	<0.001	0.723	10.896	1836	<0.001
		16	0.563	0.801	13.213	1836	<0.001	0.626	3.524	1836	<0.001
		32	0.581	0.786	10.233	1836	<0.001	0.513	3.425	1836	0.001
ND6	0.0125	4	0.847	0.801	1.677	580	0.094	0.769	2.825	580	0.005
		8	0.895	0.760	3.801	580	<0.001	0.649	6.903	580	<0.001
		16	0.956	0.735	4.959	580	<0.001	0.525	9.699	580	<0.001
		32	1.019	0.711	5.646	580	<0.001	0.383	11.667	580	<0.001
