# Supplementary material for: Evolutionary patterns in squamate mitogenomes: Are selective regimes associated with fossoriality and limblessness?
Source: Genet Mol Biol. 2026 Jul 20;49(Suppl 2):e20250226. doi: 10.1590/1678-4685-GMB-2025-0226 (PMC13384248; doi:10.1590/1678-4685-GMB-2025-0226)
Supplement: Table S3 - [file 1415-4757-GMB-49-s2-e20250226-s7.pdf]

Genetics and Molecular Biology
https://doi.org/10.1590/1678-4685-GMB-2025-0226

Supplementary Material to "Evolutionary patterns in squamate mitogenomes: are selective regimes associated with fossoriality and limblessness?"

Table S3 – Selection analyses of mitochondrial genes in selected squamate species based on PAML. (a) Results of the one-rate model (M0), in which a single ω value is shared among all species. The remaining sections show the results within each category: (b) limbless, (c) fossorial, and (d) limbless–fossorial. Abbreviations: ω (omega) = ratio of non-synonymous to synonymous substitutions (dN/dS); np = number of parameters; LnL = log-likelihood; BACK = background group; FORE = foreground group; LRT = likelihood ratio test; b_neut = relaxed branch model; b_free x b_neut = comparison between the foreground non-neutral model (b_neut) and the alternative free-ratio model (b_free); b_free x M0 = comparison between the alternative free-ratio model (b_free) and the one-rate model (M0); ln(ωF) = natural logarithm of ω estimated for foreground lineages; ln(ωB) = natural logarithm of ω estimated for background lineages. Significant p-values ($p < 0.05$) are highlighted in gray, and ln(ωF)/ln(ωB) values > 1 are shown in bold, indicating lineages in which ω is higher than in the background.

a.

	**M0**		
	omega (ω)	np	LnL
ALL	0.0814	110	-362213.844
ATP6	0.0779	110	-23285.996
ATP8	0.2925	110	-7294.318
COX1	0.0257	110	-37128.356
COX2	0.0432	110	-18363.986
COX3	0.0449	110	-20693.125
CYTB	0.0565	110	-33300.010

	M0		
	omega (ω)	np	LnL
ALL	0.0814	110	-362213.844
ATP6	0.0779	110	-23285.996
ND1	0.0480	110	-27583.924
ND2	0.0768	110	-36018.826
ND3	0.0723	110	-11630.569
ND4	0.0725	110	-45633.625
ND4L	0.0900	110	-10282.854
ND5	0.0778	110	-62362.039
ND6	0.0952	110	-19991.605

b.

	**LIMBLESS**												
	**ω (b_neut)**		**ω (b_free)**		**b_neut**		**b_free**		**b_free x b_neut**		**b_free x M0**		**LN(ωF)/LN(ωB)**
	BACK	FORE	BACK	FORE	np	LnL	np	LnL	LRT	pval	LRT	pval	
ALL	0.0694	1.0000	0.0782	0.0831	110	-391721.885	111	-362209.289	59025.190	<0.0001	9.110	0.0025	0.9761
ATP6	0.0634	1.0000	0.0684	0.0835	110	-24920.309	111	-23283.704	3273.211	<0.0001	4.585	0.0323	0.9258
ATP8	0.2869	1.0000	0.3123	0.2826	110	-7414.134	111	-7294.097	240.075	<0.0001	0.443	0.5058	1.0858
COX1	0.0196	1.0000	0.0240	0.0267	110	-43764.996	111	-37127.271	13275.450	<0.0001	2.170	0.1408	0.9712
COX2	0.0392	1.0000	0.0441	0.0428	110	-20720.345	111	-18363.941	4712.807	<0.0001	0.088	0.7662	1.0095
COX3	0.0382	1.0000	0.0416	0.0466	110	-23494.313	111	-20692.213	5604.201	<0.0001	1.824	0.1768	0.9640

CYTB	0.0431	1.0000	0.0496	0.0605	110	-36741.996	111	-33295.891	6892.211	<0.0001	8.239	0.0041	0.9339
ND1	0.0486	1.0000	0.0562	0.0443	110	-30936.308	111	-27579.060	6714.497	<0.0001	9.729	0.0018	**1.0828**
ND2	0.0801	1.0000	0.0882	0.0714	110	-38638.780	111	-36015.045	5247.471	<0.0001	7.562	0.0060	**1.0869**
ND3	0.0684	1.0000	0.0750	0.0709	110	-12504.296	111	-11630.475	1747.642	<0.0001	0.187	0.6657	1.0218
ND4	0.0735	1.0000	0.0765	0.0704	110	-49244.737	111	-45632.752	7223.971	<0.0001	1.747	0.1863	1.0323
ND4L	0.0759	1.0000	0.0908	0.0896	110	-10916.712	111	-10282.850	1267.725	<0.0001	0.009	0.9240	1.0054
ND5	0.0584	1.0000	**0.0649**	0.0858	110	-66651.690	111	-62349.096	8605.189	<0.0001	25.888	<0.0001	0.8978
ND6	0.0700	1.0000	0.0901	0.0989	110	-20788.920	111	-19991.320	1595.201	<0.0001	0.569	0.4505	0.9612

c.

	FOSSORIAL												
	**ω (b_neut)**		**ω (b_free)**		**b_neut**		**b_free**		**b_free x b_neut**		**b_free x M0**		**LN(ωF)/LN(ωB)**
	BACK	FORE	BACK	FORE	np	LnL	np	LnL	LRT	pval	LRT	pval	
ALL	0.0630	1.0000	0.0844	0.0761	110	-377291.039	111	-362201.534	30179.010	<0.0001	24.621	<0.0001	**1.0417**
ATP6	0.0615	1.0000	0.0843	0.0656	110	-24121.087	111	-23282.767	1676.640	<0.0001	6.458	0.0110	**1.1014**
ATP8	0.2475	1.0000	0.2949	0.2891	110	-7355.290	111	-7294.310	121.959	<0.0001	0.016	0.8984	1.0162
COX1	0.0191	1.0000	0.0276	0.0230	110	-40407.759	111	-37125.063	6565.392	<0.0001	6.586	0.0103	**1.0509**
COX2	0.0353	1.0000	0.0497	0.0342	110	-19610.908	111	-18356.509	2508.796	<0.0001	14.952	0.0001	**1.1248**
COX3	0.0365	1.0000	0.0485	0.0398	110	-22156.249	111	-20690.382	2931.733	<0.0001	5.485	0.0192	**1.0652**
CYTB	0.0424	1.0000	0.0561	0.0571	110	-34997.323	111	-33299.976	3394.693	<0.0001	0.068	0.7947	0.9938
ND1	0.0381	1.0000	0.0540	0.0393	110	-29249.850	111	-27575.920	3347.859	<0.0001	16.008	0.0001	**1.1084**
ND2	0.0632	1.0000	0.0863	0.0606	110	-37383.430	111	-36008.912	2749.036	<0.0001	19.827	<0.0001	**1.1446**
ND3	0.0582	1.0000	0.0797	0.0585	110	-12054.205	111	-11628.104	852.203	<0.0001	4.930	0.0264	**1.1221**
ND4	0.0603	1.0000	0.0808	0.0583	110	-47494.859	111	-45621.442	3746.834	<0.0001	24.367	<0.0001	**1.1298**
ND4L	0.0705	1.0000	0.1054	0.0661	110	-10633.954	111	-10277.570	712.769	<0.0001	10.569	0.0011	**1.2077**
ND5	0.0598	1.0000	0.0788	0.0754	110	-64487.012	111	-62361.753	4250.519	<0.0001	0.574	0.4488	1.0175
ND6	0.0769	1.0000	0.1090	0.0641	110	-20405.019	111	-19985.027	839.983	<0.0001	13.155	0.0003	**1.2394**

d.

	LIMBLESS-FOSSORIAL																		
	**ω (b_neut)**				**ω (b_free)**				**b_neut**		**b_free**		**b_free x b_neut**		**b_free x M0**		**LN(ωF)/LN(ωB)**		
	BACK	LIMB	FOSS	L&F	BACK	LIMB	FOSS	L&F	np	LnL	np	LnL	LRT	pval	D	pval	LIMB	FOSS	L&F
ALL	0.0727	0.0588	0.0632	1.0000	0.0790	0.0911	0.0619	0.0766	112	-366407.304	113	-362180.930	8452.747	<0.0001	65.828	<0.0001	0.9439	**1.0962**	**1.0123**
ATP6	0.0658	0.0625	0.0536	1.0000	0.0693	0.1025	0.0452	0.0663	112	-24111.726	113	-23275.666	1672.120	<0.0001	20.660	0.0001	0.8533	**1.1603**	**1.0167**
ATP8	0.3156	0.2051	0.0470	1.0000	0.3358	0.2483	0.0568	0.3094	112	-7343.092	113	-7289.617	106.951	<0.0001	9.403	0.0244	**1.2766**	**2.6279**	**1.0750**
COX1	0.0212	0.0181	0.0148	1.0000	0.0241	0.0328	0.0167	0.0231	112	-40388.872	113	-37118.712	6540.319	<0.0001	19.288	0.0002	0.9166	**1.0986**	**1.0106**
COX2	0.0406	0.0337	0.0320	1.0000	0.0441	0.0562	0.0375	0.0340	112	-19594.700	113	-18354.382	2480.636	<0.0001	19.208	0.0002	0.9228	**1.0526**	**1.0838**
COX3	0.0404	0.0344	0.0402	1.0000	0.0417	0.0570	0.0350	0.0397	112	-22148.478	113	-20685.669	2925.619	<0.0001	14.912	0.0019	0.9015	**1.0544**	**1.0152**
CYTB	0.0432	0.0429	0.1020	1.0000	0.0481	0.0658	0.0887	0.0563	112	-34988.614	113	-33292.101	3393.028	<0.0001	15.819	0.0012	0.8965	0.7982	0.9481
ND1	0.0501	0.0308	0.0684	1.0000	0.0553	0.0518	0.0919	0.0387	112	-29232.599	113	-27573.972	3317.253	<0.0001	19.904	0.0002	**1.0225**	0.8242	**1.1228**
ND2	0.0829	0.0545	0.0893	1.0000	0.0875	0.0847	0.0886	0.0596	112	-37368.180	113	-36008.151	2720.059	<0.0001	21.350	0.0001	**1.0136**	0.9948	**1.1577**
ND3	0.0706	0.0526	0.0753	1.0000	0.0745	0.0849	0.0758	0.0577	112	-12049.408	113	-11627.655	843.506	<0.0001	5.827	0.1203	0.9496	0.9933	1.0980
ND4	0.0740	0.0533	0.0792	1.0000	0.0769	0.0850	0.0673	0.0578	112	-47467.267	113	-45620.368	3693.797	<0.0001	26.513	<0.0001	0.9610	**1.0517**	**1.1112**
ND4L	0.0772	0.0720	0.0406	1.0000	0.0963	0.1170	0.0409	0.0684	112	-10625.684	113	-10276.272	698.823	<0.0001	13.164	0.0043	0.9171	**1.3665**	**1.1462**
ND5	0.0610	0.0633	0.0711	1.0000	0.0644	0.0962	0.0686	0.0753	112	-64459.577	113	-62342.366	4234.422	<0.0001	39.348	<0.0001	0.8539	0.9772	0.9434
ND6	0.0754	0.0917	0.0578	1.0000	0.0926	0.1307	0.0650	0.0643	112	-20389.098	113	-19981.889	814.419	<0.0001	19.432	0.0002	0.8552	**1.1487**	**1.1532**
