# Supplementary material for: Evolutionary patterns in squamate mitogenomes: Are selective regimes associated with fossoriality and limblessness?
Source: Genet Mol Biol. 2026 Jul 20;49(Suppl 2):e20250226. doi: 10.1590/1678-4685-GMB-2025-0226 (PMC13384248; doi:10.1590/1678-4685-GMB-2025-0226)
Supplement: Table S4 - [file 1415-4757-GMB-49-s2-e20250226-s8.pdf]

Genetics and Molecular Biology

https://doi.org/10.1590/1678-4685-GMB-2025-0226

Supplementary Material to "Evolutionary patterns in squamate mitogenomes: are selective regimes associated with fossoriality and limblessness?"

Table S4 – Selection analyses of mitochondrial genes in selected squamate species based on PAML and ETE3 lineage-specific ω estimates. (a) Results of the one-rate model (M0), in which a single ω value is shared among all species, and (b) the neutral one-rate model (M0, ω = 1), in which all species are constrained to have ω = 1. The remaining sections show the results within each ecological category: (c) limbless and (d) fossorial. Abbreviations: ω (omega) = ratio of non-synonymous to synonymous substitutions (dN/dS); np = number of parameters; LnL = log-likelihood; BACK = background group; LRT = likelihood ratio test; b_free = alternative model; ln(ωF) = natural logarithm of ω estimated for foreground lineages; ln(ωB) = natural logarithm of ω estimated for background lineages; Pegregius = *Plestiodon egregius*. Significant p-values ($p < 0.05$) are highlighted in gray, and ln(ωF)/ln(ωB) values > 1 are shown in bold, indicating lineages in which ω is higher than in the background.

a.

	**M0**		
	ω	np	LnL
ALL	0.0814	110	-362213.844
ATP6	0.0779	110	-23285.996
ATP8	0.2925	110	-7294.318
COX1	0.0257	110	-37128.356
COX2	0.0432	110	-18363.986
COX3	0.0449	110	-20693.125

b.

	**M0 (ω = 1)**				
	ω	np	LnL	LRT	pval
ALL	1.0000	109	-409525.950	94624.210	<0.0001
ATP6	1.0000	109	-25910.314	5248.636	<0.0001
ATP8	1.0000	109	-7470.471	352.305	<0.0001
COX1	1.0000	109	-47698.641	21140.570	<0.0001
COX2	1.0000	109	-22014.304	7300.638	<0.0001
COX3	1.0000	109	-25159.226	8932.202	<0.0001

CYTB	0.0565	110	-33300.010
ND1	0.0480	110	-27583.924
ND2	0.0768	110	-36018.826
ND3	0.0723	110	-11630.569
ND4	0.0725	110	-45633.625
ND4L	0.0900	110	-10282.854
ND5	0.0778	110	-62362.039
ND6	0.0952	110	-19991.605

CYTB	1.0000	109	-38961.215	11322.409	<0.0001
ND1	1.0000	109	-32721.554	10275.259	<0.0001
ND2	1.0000	109	-39994.038	7950.424	<0.0001
ND3	1.0000	109	-13012.318	2763.500	<0.0001
ND4	1.0000	109	-51291.055	11314.859	<0.0001
ND4L	1.0000	109	-11322.576	2079.444	<0.0001
ND5	1.0000	109	-69572.705	14421.331	<0.0001
ND6	1.0000	109	-21360.994	2738.779	<0.0001

c.

	LIMBLESS													
	**ω (b_free)**					**b_free x M0**				**LN(ωF)/LN(ωB)**				
	BACK	Amphisbaenia	*Isopachys*	Serpentes	Anguinae	np	LnL	LRT	pval	Amphisbaenia	*Isopachys*	Serpentes	Anguinae
ALL	0.0785	**0.0738**	**0.0503**	0.0880	0.0833	114	-362174.324	79.040	<0.0001	**1.0242**	**1.1750**	0.9549	0.9764
ATP6	0.0680	**0.0602**	**0.0080**	0.0842	0.1060	114	-23273.609	24.774	0.0001	**1.0451**	**1.7947**	0.9203	0.8349
ATP8	0.3176	**0.2823**	**0.0339**	0.3326	**0.2113**	114	-7290.095	8.446	0.0765	1.1025	2.9500	0.9598	1.3552
COX1	0.0241	**0.0199**	**0.0160**	0.0326	**0.0230**	114	-37111.030	34.652	<0.0001	**1.0511**	**1.1100**	0.9188	**1.0129**
COX2	0.0438	**0.0312**	**0.0361**	0.0455	0.0550	114	-18358.236	11.500	0.0215	**1.1080**	**1.0616**	0.9878	0.9269
COX3	0.0413	**0.0340**	**0.0310**	0.0506	0.0580	114	-20684.026	18.198	0.0011	**1.0610**	**1.0905**	0.9361	0.8935
CYTB	0.0500	**0.0512**	**0.0291**	0.0662	0.0604	114	-33289.161	21.699	0.0002	0.9919	**1.1802**	0.9061	0.9366
ND1	0.0561	**0.0378**	**0.0306**	**0.0436**	0.0589	114	-27573.631	20.586	0.0004	**1.1375**	**1.2107**	**1.0874**	0.9831
ND2	0.0879	**0.0415**	**0.0221**	0.0810	0.0773	114	-35999.454	38.743	<0.0001	**1.3088**	**1.5673**	**1.0337**	**1.0531**
ND3	0.0751	**0.0383**	**0.0351**	0.0843	0.0731	114	-11623.534	14.070	0.0071	**1.2599**	**1.2939**	0.9553	**1.0104**
ND4	0.0763	**0.0533**	**0.0366**	**0.0710**	0.0928	114	-45620.005	27.241	<0.0001	**1.1396**	**1.2859**	**1.0279**	0.9238
ND4L	0.0921	**0.0548**	**0.0101**	0.1036	0.1125	114	-10271.854	22.001	0.0002	**1.2173**	**1.9286**	0.9506	0.9158
ND5	**0.0645**	**0.0649**	**0.0440**	0.0889	0.0971	114	-62338.990	46.098	<0.0001	0.9978	**1.1393**	0.8832	0.8509
ND6	**0.0904**	**0.0420**	**0.0151**	**0.0898**	0.1654	114	-19973.670	35.869	<0.0001	**1.3191**	**1.7457**	**1.0031**	0.7489

d.

	FOSSORIAL														
	**ω (b_free)**											**b_free x M0**			
					Serpentes										
	BACK	Amphisbaenia	*Isopachys*	Pegregius	Scolecophidia	*Anilius*	*Cylindrophis*	*Xenopeltis*	*Achalinus*	*Micrurus*	*Calamaria*	np	LnL	LRT	pval
ALL	0.0846	0.0738	**0.0496**	0.0606	0.0775	0.0668	0.0689	0.0596	0.0962	0.0780	0.0939	120	-362156.500	114.690	<0.0001
ATP6	0.0841	**0.0601**	**0.0073**	**0.0429**	**0.0493**	**0.0446**	**0.0451**	**0.0294**	0.1217	0.1398	**0.0567**	120	-23264.660	42.672	<0.0001
ATP8	0.3025	**0.2802**	**0.1003**	**0.0636**	0.4794	0.5044	**0.2311**	**0.1273**	0.4163	0.3270	**0.0058**	120	-7283.279	22.078	0.0147
COX1	0.0279	**0.0199**	**0.0159**	**0.0162**	0.0337	**0.0239**	**0.0225**	**0.0166**	0.0293	**0.0214**	0.0358	120	-37115.173	26.367	0.0033
COX2	0.0489	**0.0310**	**0.0354**	**0.0367**	**0.0168**	0.0468	0.0508	**0.0136**	0.0436	0.0991	0.0607	120	-18343.570	40.832	<0.0001
COX3	0.0486	0.0342	0.0285	0.0319	0.0409	0.0394	**0.0179**	0.0261	0.0593	0.0660	0.0618	120	-20680.006	26.237	0.0034
CYTB	0.0558	0.0510	**0.0290**	0.0817	0.0549	0.0587	0.0690	0.0466	0.0719	0.0879	0.0804	120	-33291.706	16.608	0.0835
ND1	0.0539	0.0379	0.0306	0.0956	0.0443	0.0255	**0.0248**	0.0506	0.0383	0.0287	0.0628	120	-27569.547	28.754	0.0014
ND2	0.0857	0.0416	**0.0220**	0.0872	0.0489	0.0343	0.0430	0.0403	0.1140	0.0848	0.0755	120	-35986.256	65.140	<0.0001
ND3	0.0784	0.0383	0.0348	0.0733	0.0664	0.0329	0.0436	0.1109	0.1173	0.1782	**0.0265**	120	-11618.226	24.685	0.0060
ND4	0.0807	0.0533	0.0358	0.0656	0.0367	**0.0232**	0.0561	0.0444	0.0954	0.0690	0.0662	120	-45603.902	59.446	<0.0001
ND4L	0.1072	0.0547	0.0117	0.0448	0.1121	0.1347	0.0784	0.0293	0.1627	0.0380	**0.0016**	120	-10258.273	49.163	<0.0001
ND5	0.0783	0.0654	0.0438	0.0638	**0.0391**	0.0480	0.0778	0.0666	0.1240	0.1023	0.0843	120	-62336.366	51.348	<0.0001
ND6	0.1096	0.0422	0.0168	0.0707	0.0932	0.0313	**0.0076**	0.0648	0.0983	0.0699	0.1601	120	-19976.244	30.721	0.0007

d.

	FOSSORIAL (cont.)									
	LN(ωF)/LN(ωB)									
				Serpentes						
	Amphisbaenia	*Isopachys*	Pegregius	Scolecophidia	*Anilius*	*Cylindrophis*	*Xenopeltis*	*Achalinus*	*Micrurus*	*Calamaria*
ALL	**1.0556**	**1.2167**	**1.1350**	**1.0357**	**1.0959**	**1.0835**	**1.1418**	0.9480	**1.0333**	0.9580
ATP6	**1.1357**	**1.9875**	**1.2724**	**1.2161**	**1.2564**	**1.2521**	**1.4243**	0.8510	0.7950	**1.1592**
ATP8	**1.0640**	**1.9237**	**2.3041**	0.6149	0.5724	**1.2253**	**1.7239**	0.7330	0.9350	**4.3116**
COX1	**1.0941**	**1.1570**	**1.1523**	0.9469	**1.0428**	**1.0596**	**1.1451**	0.9855	**1.0735**	0.9303
COX2	**1.1509**	**1.1074**	**1.0952**	**1.3548**	**1.0145**	0.9872	**1.4238**	**1.0381**	0.7659	0.9285
COX3	**1.1158**	**1.1767**	**1.1388**	**1.0568**	**1.0691**	**1.3294**	**1.2050**	0.9340	0.8989	0.9206
CYTB	1.0315	1.2273	0.8682	1.0055	0.9824	0.9263	1.0629	0.9124	0.8428	0.8735
ND1	**1.1207**	**1.1935**	0.8036	**1.0670**	**1.2562**	**1.2662**	**1.0217**	**1.1170**	**1.2162**	0.9473
ND2	**1.2940**	**1.5540**	0.9932	**1.2287**	**1.3725**	**1.2813**	**1.3077**	0.8840	**1.0042**	**1.0516**
ND3	**1.2814**	**1.3195**	**1.0263**	**1.0651**	**1.3407**	**1.2300**	0.8638	0.8417	0.6774	**1.4263**
ND4	**1.1652**	**1.3228**	**1.0824**	**1.3127**	**1.4948**	**1.1448**	**1.2378**	0.9336	**1.0622**	**1.0786**
ND4L	**1.3013**	**1.9909**	**1.3907**	0.9801	0.8977	**1.1402**	**1.5809**	0.8133	**1.4645**	**2.8944**
ND5	**1.0705**	**1.2282**	**1.0806**	**1.2725**	**1.1923**	**1.0025**	**1.0634**	0.8194	0.8952	0.9713
ND6	**1.4312**	**1.8491**	**1.1981**	**1.0732**	**1.5666**	**2.2086**	**1.2378**	**1.0493**	**1.2030**	0.8285
