# Supplementary material for: Evolutionary patterns in squamate mitogenomes: Are selective regimes associated with fossoriality and limblessness?
Source: Genet Mol Biol. 2026 Jul 20;49(Suppl 2):e20250226. doi: 10.1590/1678-4685-GMB-2025-0226 (PMC13384248; doi:10.1590/1678-4685-GMB-2025-0226)
Supplement: Table S5 - [file 1415-4757-GMB-49-s2-e20250226-s9.pdf]

Genetics and Molecular Biology
https://doi.org/10.1590/1678-4685-GMB-2025-0226

Supplementary Material to "Evolutionary patterns in squamate mitogenomes: are selective regimes associated with fossoriality and limblessness?"

Table S5 – Selection analyses of mitochondrial genes were performed using branch-site models in selected squamate lineages, with lineage-specific ω estimates obtained using PAML and ETE3. (a) Results of the null model (M1); model bsC for (b) limbless, (c) fossorial, and (d) limbless–fossorial lineages; (e) null model (M3) and model bsD for (f) limbless, (g) fossorial, and (h) limbless–fossorial lineages. Abbreviations: Proportion = proportion of sites in each ω category; ω (omega) = ratio of non-synonymous to synonymous substitutions (dN/dS); np = number of parameters; LnL = log-likelihood; BACK = background group; LRT = likelihood ratio test; b_free = alternative model; Pegregius = Plestiodon egregius; ln(ωF) = natural logarithm of ω estimated for limbless lineages; ln(ωB) = natural logarithm of ω estimated for background lineages. Significant p-values ($p < 0.05$) are highlighted in gray, and ln(ωF)/ln(ωB) values > 1 are shown in bold, indicating lineages in which ω is higher than in the background.

a.

	M1					
	proportion		ω		np	LnL
ALL	**0.897**	0.103	0.0782	1.0000	111	-358205.762
ATP6	**0.951**	0.049	0.0815	1.0000	111	-23214.111
ATP8	0.454	**0.546**	0.1538	1.0000	111	-7112.093
COX1	**0.990**	0.010	0.0248	1.0000	111	-37057.760
COX2	**0.976**	0.024	0.0414	1.0000	111	-18292.723
COX3	**0.941**	0.059	0.0433	1.0000	111	-20596.658
CYTB	**0.950**	0.050	0.0548	1.0000	111	-33082.075
ND1	**0.954**	0.046	0.0458	1.0000	111	-27373.498
ND2	**0.960**	0.040	0.0782	1.0000	111	-35893.254
ND3	**0.843**	0.157	0.0732	1.0000	111	-11503.941
ND4	**0.926**	0.074	0.0752	1.0000	111	-45423.752
ND4L	**0.978**	0.022	0.0943	1.0000	111	-10281.697
ND5	**0.895**	0.105	0.0825	1.0000	111	-61934.918
ND6	**0.727**	0.273	0.1051	1.0000	111	-19685.411

b.

	LIMBLESS (bsC)													
	proportion			ω						Alternative		M1 *x* bsC		LN(ωF)/LN(ωB)
				background			foreground			np	LnL	LRT	pval	
ALL	0.483	0.035	0.482	0.0139	1.0000	0.1473	0.0139	1.0000	0.1504	114	-348590.057	19231.411	<0.0001	0.9890
ATP6	0.458	0.009	0.533	0.0153	1.0000	0.1254	0.0153	1.0000	0.1387	114	-22565.676	1296.870	<0.0001	0.9516
ATP8	0.214	0.368	0.419	0.0384	1.0000	0.3565	0.0384	1.0000	0.2687	114	-7031.927	160.332	<0.0001	**1.2741**
COX1	0.658	0.003	0.339	0.0032	1.0000	0.0693	0.0032	1.0000	0.0714	114	-35995.710	2124.101	<0.0001	0.9889
COX2	0.478	0.022	0.500	0.0059	1.0000	0.0772	0.0059	1.0000	0.0750	114	-17850.824	883.797	<0.0001	**1.0116**
COX3	0.637	0.008	0.355	0.0082	1.0000	0.1129	0.0082	1.0000	0.1105	114	-19901.595	1390.127	<0.0001	**1.0099**
CYTB	0.531	0.016	0.453	0.0081	1.0000	0.0986	0.0081	1.0000	0.1178	114	-31998.733	2166.686	<0.0001	0.9234
ND1	0.507	0.032	0.461	0.0065	1.0000	0.1007	0.0065	1.0000	0.0841	114	-26621.232	1504.532	<0.0001	**1.0784**
ND2	0.455	0.015	0.531	0.0229	1.0000	0.1471	0.0229	1.0000	0.1191	114	-35136.672	1513.164	<0.0001	**1.1104**
ND3	0.589	0.010	0.401	0.0194	1.0000	0.1720	0.0194	1.0000	0.1475	114	-11177.170	653.542	<0.0001	**1.0873**
ND4	0.418	0.012	0.569	0.0132	1.0000	0.1224	0.0132	1.0000	0.1136	114	-44355.579	2136.345	<0.0001	**1.0352**
ND4L	0.651	0.000	0.349	0.0449	1.0000	0.1840	0.0449	1.0000	0.1648	114	-10108.189	347.015	<0.0001	**1.0650**
ND5	0.449	0.018	0.533	0.0166	1.0000	0.1155	0.0166	1.0000	0.1440	114	-60253.689	3362.458	<0.0001	0.8978
ND6	0.469	0.061	0.470	0.0351	1.0000	0.1571	0.0351	1.0000	0.1895	114	-19366.274	638.274	<0.0001	0.8985

c.

	FOSSORIAL (bsC)														
	proportion			ω						Alternative		M1 x bsC		LN(ωF)/LN(ωB)
				background			foreground			np	LnL	LRT	pval	
ALL	0.483	0.035	0.482	0.0138	1.0000	0.1521	0.0138	1.0000	0.1447	114	-348587.560	19236.406	<0.0001	**1.0266**
ATP6	0.456	0.009	0.535	0.0147	1.0000	0.1424	0.0147	1.0000	0.1172	114	-22563.913	1300.397	<0.0001	**1.1001**
ATP8	0.212	0.376	0.413	0.0381	1.0000	0.3153	0.0381	1.0000	0.2609	114	-7032.802	158.581	<0.0001	**1.1640**
COX1	0.659	0.004	0.338	0.0032	1.0000	0.0754	0.0032	1.0000	0.0636	114	-35993.225	2129.071	<0.0001	**1.0656**
COX2	0.475	0.022	0.503	0.0056	1.0000	0.0861	0.0056	1.0000	0.0603	114	-17844.030	897.385	<0.0001	**1.1457**
COX3	0.634	0.008	0.358	0.0079	1.0000	0.1220	0.0079	1.0000	0.0941	114	-19897.255	1398.807	<0.0001	**1.1237**
CYTB	0.533	0.015	0.452	0.0082	1.0000	0.1088	0.0082	1.0000	0.1155	114	-32001.483	2161.184	<0.0001	0.9728
ND1	0.507	0.032	0.461	0.0065	1.0000	0.0954	0.0065	1.0000	0.0808	114	-26621.711	1503.573	<0.0001	**1.0705**
ND2	0.452	0.015	0.534	0.0223	1.0000	0.1372	0.0223	1.0000	0.1117	114	-35136.798	1512.913	<0.0001	**1.1034**
ND3	0.584	0.012	0.404	0.0191	1.0000	0.1578	0.0191	1.0000	0.1477	114	-11177.665	652.554	<0.0001	**1.0359**
ND4	0.414	0.012	0.574	0.0126	1.0000	0.1238	0.0126	1.0000	0.1029	114	-44351.990	2143.523	<0.0001	**1.0886**
ND4L	0.644	0.000	0.356	0.0441	1.0000	0.1832	0.0441	1.0000	0.1505	114	-10107.388	348.617	<0.0001	**1.1156**
ND5	0.449	0.018	0.533	0.0165	1.0000	0.1334	0.0165	1.0000	0.1329	114	-60262.523	3344.790	<0.0001	**1.0020**
ND6	0.468	0.061	0.471	0.0343	1.0000	0.1792	0.0343	1.0000	0.1702	114	-19367.775	635.273	<0.0001	**1.0301**

	FOSSORIAL - LIMBLESS (bsC)															
	proportion			ω												
				background			limbless			fossorial			fossorial-limbless			
ALL	0.483	0.035	0.482	0.0138	1.0000	0.1491	0.0138	1.0000	0.1563	0.0138	1.0000	0.1073	0.0138	1.0000	0.1459
ATP6	0.456	0.009	0.535	0.0146	1.0000	0.1255	0.0146	1.0000	0.1640	0.0146	1.0000	0.0831	0.0146	1.0000	0.1174
ATP8	0.214	0.362	0.424	0.0385	1.0000	0.3751	0.0385	1.0000	0.2658	0.0385	1.0000	0.1364	0.0385	1.0000	0.2729
COX1	0.658	0.004	0.339	0.0032	1.0000	0.0695	0.0032	1.0000	0.0832	0.0032	1.0000	0.0527	0.0032	1.0000	0.0638
COX2	0.474	0.022	0.504	0.0055	1.0000	0.0758	0.0055	1.0000	0.0978	0.0055	1.0000	0.0880	0.0055	1.0000	0.0593
COX3	0.636	0.008	0.357	0.0079	1.0000	0.1117	0.0079	1.0000	0.1350	0.0079	1.0000	0.1116	0.0079	1.0000	0.0938
CYTB	0.531	0.016	0.453	0.0080	1.0000	0.0967	0.0080	1.0000	0.1225	0.0080	1.0000	0.1435	0.0080	1.0000	0.1139
ND1	0.507	0.032	0.461	0.0065	1.0000	0.1005	0.0065	1.0000	0.0890	0.0065	1.0000	0.1059	0.0065	1.0000	0.0807
ND2	0.453	0.015	0.532	0.0225	1.0000	0.1465	0.0225	1.0000	0.1277	0.0225	1.0000	0.1309	0.0225	1.0000	0.1119
ND3	0.590	0.009	0.400	0.0196	1.0000	0.1730	0.0196	1.0000	0.1449	0.0196	1.0000	0.1733	0.0196	1.0000	0.1500
ND4	0.414	0.012	0.574	0.0126	1.0000	0.1228	0.0126	1.0000	0.1251	0.0126	1.0000	0.0952	0.0126	1.0000	0.1032
ND4L	0.646	0.000	0.354	0.0442	1.0000	0.1844	0.0442	1.0000	0.1826	0.0442	1.0000	0.1311	0.0442	1.0000	0.1518
ND5	0.447	0.018	0.535	0.0161	1.0000	0.1148	0.0161	1.0000	0.1565	0.0161	1.0000	0.1060	0.0161	1.0000	0.1325
ND6	0.470	0.061	0.469	0.0350	1.0000	0.1608	0.0350	1.0000	0.2065	0.0350	1.0000	0.0944	0.0350	1.0000	0.1768

d.

	FOSSORIAL - LIMBLESS (bsC)						
	Alternative		M1 x bsC		LN(ωF)/LN(ωB)		
	np	LnL	LRT	pval	limbless	fossorial	fossorial-limbless
ALL	116	-348580.320	19250.885	<0.0001	0.9753	**1.1726**	**1.0112**
ATP6	116	-22560.195	1307.833	<0.0001	0.8711	**1.1985**	**1.0321**
ATP8	116	-7030.755	162.675	<0.0001	**1.3514**	**2.0315**	**1.3244**
COX1	116	-35991.273	2132.974	<0.0001	0.9323	**1.1037**	**1.0321**
COX2	116	-17841.374	902.697	<0.0001	0.9013	0.9422	**1.0955**
COX3	116	-19895.691	1401.935	<0.0001	0.9134	**1.0002**	**1.0797**
CYTB	116	-31997.665	2168.821	<0.0001	0.8990	0.8313	0.9301
ND1	116	-26620.710	1505.576	<0.0001	**1.0528**	0.9774	**1.0952**
ND2	116	-35135.406	1515.696	<0.0001	**1.0714**	**1.0584**	**1.1400**
ND3	116	-11177.148	653.586	<0.0001	**1.1012**	0.9989	**1.0812**
ND4	116	-44351.905	2143.694	<0.0001	0.9909	**1.1214**	**1.0826**
ND4L	116	-10107.348	348.698	<0.0001	**1.0060**	**1.2017**	**1.1150**
ND5	116	-60250.192	3369.452	<0.0001	0.8569	**1.0372**	0.9339
ND6	116	-19364.679	641.464	<0.0001	0.8631	**1.2915**	0.9480

e.

	M3							
	proportion			ω			np	LnL
ALL	0.384	0.394	0.222	0.0040	0.0607	0.1809	114	-346694.480
ATP6	0.293	0.394	0.313	0.0288	0.2066	0.6089	114	-22456.478
ATP8	0.208	0.359	0.433	0.0055	0.0801	0.2585	114	-7021.184
COX1	0.581	0.246	0.173	0.0014	0.0327	0.1085	114	-35899.910
COX2	0.420	0.443	0.137	0.0032	0.0465	0.1631	114	-17733.533
COX3	0.501	0.304	0.196	0.0030	0.0442	0.1681	114	-19781.935
CYTB	0.478	0.363	0.158	0.0050	0.0679	0.2035	114	-31828.954
ND1	0.433	0.409	0.158	0.0032	0.0542	0.1839	114	-26466.056
ND2	0.234	0.417	0.348	0.0052	0.0572	0.1627	114	-34998.533
ND3	0.305	0.409	0.286	0.0026	0.0460	0.1935	114	-11089.442
ND4	0.298	0.463	0.239	0.0043	0.0620	0.1973	114	-44031.383
ND4L	0.175	0.542	0.283	0.0083	0.0641	0.1880	114	-10055.820
ND5	0.297	0.412	0.291	0.0050	0.0630	0.1964	114	-59897.962
ND6	0.206	0.460	0.334	0.0114	0.0663	0.2523	114	-19260.710

f.

	LIMBLESS (bsD)													
	proportion			ω						Alternative		M3 x bsD		LN(ωF)/LN(ωB)
				background			foreground			np	LnL	LRT	pval	
ALL	0.377	0.415	0.208	9.0000	0.0100	0.2683	0.0763	0.0100	0.2146	115	-346806.374	223.789	<0.0001	**1.1697**
ATP6	0.382	0.358	0.260	0.0763	0.0100	0.1806	0.0138	0.1673	0.5413	115	-22464.933	16.909	<0.0001	0.3586
ATP8	0.145	0.381	0.474	0.0138	0.1673	0.6191	0.0898	0.0100	0.2784	115	-7021.676	0.984	0.3213	2.6663
COX1	0.080	0.697	0.223	0.1713	0.0100	0.0694	0.1713	0.0100	0.0715	115	-36039.113	278.407	<0.0001	0.9891
COX2	0.390	0.486	0.123	0.0594	0.0100	0.1930	0.0594	0.0100	0.1852	115	-17761.842	56.617	<0.0001	**1.0249**
COX3	0.244	0.595	0.161	0.0662	0.0100	0.1906	0.0662	0.0100	0.2009	115	-19810.336	56.803	<0.0001	0.9684
CYTB	0.478	0.363	0.159	0.0050	0.0678	0.1973	0.0050	0.0678	0.2060	115	-31828.830	0.247	0.6189	0.9734
ND1	0.432	0.407	0.161	0.0032	0.0538	0.2082	0.0032	0.0538	0.1699	115	-26463.387	5.338	0.0209	**1.1297**
ND2	0.405	0.271	0.325	0.0643	0.0100	0.1864	0.0643	0.0100	0.1657	115	-35006.203	15.340	0.0001	**1.0703**
ND3	0.344	0.392	0.264	0.0607	0.0100	0.2699	0.0607	0.0100	0.1916	115	-11095.889	12.895	0.0003	**1.2614**
ND4	0.299	0.237	0.464	0.0043	0.1982	0.0659	0.0043	0.1982	0.0603	115	-44030.435	1.896	0.1686	1.0326
ND4L	0.175	0.283	0.542	0.0083	0.1880	0.0642	0.0083	0.1880	0.0641	115	-10055.820	0.000	0.9894	1.0007
ND5	0.411	0.342	0.247	0.0764	0.0100	0.1906	0.0764	0.0100	0.2378	115	-59911.135	26.347	<0.0001	0.8663
ND6	0.199	0.442	0.359	0.0110	0.0637	0.2135	0.0110	0.0637	0.2583	115	-19259.867	1.685	0.1942	0.8766

g.

	FOSSORIAL (bsD)													
	proportion			ω						Alternative		M3 x bsD		LN(ωF)/LN(ωB)
				background			foreground			np	LnL	LRT	pval	
ALL	0.378	0.415	0.207	0.0901	0.0100	0.2680	0.0901	0.0100	0.2874	115	-346802.913	216.866	<0.0001	0.9471
ATP6	0.246	0.364	0.390	0.2041	0.0100	0.0863	0.2041	0.0100	0.0665	115	-22462.284	11.611	0.0007	**1.1066**
ATP8	0.367	0.209	0.424	0.2106	0.0290	0.5789	0.2106	0.0290	0.6912	115	-7020.567	1.234	0.2667	0.6755
COX1	0.219	0.696	0.084	0.0699	0.0100	0.1731	0.0699	0.0100	0.1603	115	-36038.972	278.124	<0.0001	**1.0439**
COX2	0.389	0.486	0.125	0.0590	0.0100	0.1969	0.0590	0.0100	0.1715	115	-17761.223	55.380	<0.0001	**1.0853**
COX3	0.501	0.304	0.195	0.0030	0.0442	0.1779	0.0030	0.0442	0.1549	115	-19780.918	2.033	0.1539	1.0800
CYTB	0.339	0.512	0.149	0.0797	0.0100	0.2100	0.0797	0.0100	0.2434	115	-31851.967	46.027	<0.0001	0.9055
ND1	0.138	0.504	0.357	0.2106	0.0100	0.0692	0.2106	0.0100	0.0670	115	-26501.552	70.991	<0.0001	**1.0124**
ND2	0.405	0.271	0.325	0.0642	0.0100	0.1740	0.0642	0.0100	0.1700	115	-35007.390	17.714	<0.0001	**1.0133**
ND3	0.337	0.388	0.276	0.0587	0.0100	0.2173	0.0587	0.0100	0.2028	115	-11098.648	18.412	<0.0001	**1.0454**
ND4	0.299	0.233	0.468	0.0043	0.1983	0.0671	0.0043	0.1983	0.0565	115	-44027.732	7.302	0.0069	**1.0634**
ND4L	0.282	0.184	0.534	0.1886	0.0100	0.0721	0.1886	0.0100	0.0574	115	-10054.524	2.591	0.1075	1.0872
ND5	0.241	0.345	0.413	0.2215	0.0100	0.0781	0.2215	0.0100	0.0745	115	-59918.007	40.090	<0.0001	**1.0183**
ND6	0.453	0.203	0.344	0.0654	0.0113	0.2405	0.0654	0.0113	0.2615	115	-19260.517	0.385	0.5350	0.9413

h.

	FOSSORIAL-LIMBLESS (bsD)															
	proportion			ω												
				background			limbless			fossorial			fossorial-limbless			
ALL	0.378	0.415	0.208	0.0902	0.0100	0.2718	0.0902	0.0100	0.2638	0.0902	0.0100	0.2136	0.0902	0.0100	0.2898
ATP6	0.377	0.357	0.266	0.0754	0.0100	0.1798	0.0754	0.0100	0.2179	0.0754	0.0100	0.1271	0.0754	0.0100	0.2099
ATP8	0.143	0.294	0.563	0.0130	0.1379	0.6710	0.0130	0.1379	0.3394	0.0130	0.1379	0.0932	0.0130	0.1379	0.5652
COX1	0.575	0.181	0.244	0.0013	0.1069	0.0260	0.0013	0.1069	0.0336	0.0013	0.1069	0.0165	0.0013	0.1069	0.0342
COX2	0.387	0.485	0.128	0.0586	0.0100	0.1881	0.0586	0.0100	0.2048	0.0586	0.0100	0.1707	0.0586	0.0100	0.1693
COX3	0.238	0.593	0.168	0.0641	0.0100	0.1879	0.0641	0.0100	0.2219	0.0641	0.0100	0.1223	0.0641	0.0100	0.1823
CYTB	0.339	0.512	0.149	0.0797	0.0100	0.2139	0.0797	0.0100	0.2053	0.0797	0.0100	0.3651	0.0797	0.0100	0.2403
ND1	0.433	0.408	0.159	0.0032	0.0537	0.2073	0.0032	0.0537	0.1883	0.0032	0.0537	0.2666	0.0032	0.0537	0.1592
ND2	0.323	0.273	0.403	0.1705	0.0100	0.0672	0.1705	0.0100	0.0764	0.1705	0.0100	0.1208	0.1705	0.0100	0.0546
ND3	0.360	0.401	0.240	0.0657	0.0100	0.3229	0.0657	0.0100	0.1594	0.0657	0.0100	0.1279	0.0657	0.0100	0.2143
ND4	0.299	0.233	0.468	0.0042	0.1978	0.0659	0.0042	0.1978	0.0686	0.0042	0.1978	0.0608	0.0042	0.1978	0.0562
ND4L	0.175	0.288	0.537	0.0082	0.1852	0.0654	0.0082	0.1852	0.0775	0.0082	0.1852	0.0337	0.0082	0.1852	0.0564
ND5	0.301	0.277	0.422	0.0052	0.2003	0.0587	0.0052	0.2003	0.0765	0.0052	0.2003	0.0482	0.0052	0.2003	0.0634
ND6	0.441	0.198	0.361	0.0639	0.0111	0.2160	0.0639	0.0111	0.2492	0.0639	0.0111	0.1643	0.0639	0.0111	0.2673

h.

	FOSSORIAL-LIMBLESS (bsD)						
	Alternative				LN(ωF)/LN(ωB)		
	np	LnL	LRT	pval	limbless	fossorial	fossorial-limbless
ALL	117	-346798.497	208.034	<0.0001	**1.0230**	**1.1852**	0.9510
ATP6	117	-22464.696	16.436	0.0009	0.8878	**1.2019**	0.9096
ATP8	117	-7012.104	18.161	0.0004	2.7084	**5.9472**	1.4302
COX1	117	-35897.405	5.010	0.1711	0.9295	1.1244	0.9253
COX2	117	-17761.082	55.097	<0.0001	0.9489	**1.0581**	**1.0628**
COX3	117	-19808.770	53.670	<0.0001	0.9005	**1.2569**	**1.0179**
CYTB	117	-31851.160	44.413	<0.0001	**1.0265**	0.6532	0.9243
ND1	117	-26462.312	7.489	0.0578	1.0610	0.8402	1.1679
ND2	117	-34999.511	1.956	0.5816	0.9528	0.7830	1.0769
ND3	117	-11093.891	8.898	0.0307	**1.6242**	**1.8193**	**1.3625**
ND4	117	-44027.588	7.590	0.0553	0.9853	1.0293	1.0581
ND4L	117	-10053.426	4.787	0.1881	0.9379	1.2426	1.0542
ND5	117	-59891.922	12.080	0.0071	0.9068	**1.0697**	0.9728
ND6	117	-19259.511	2.397	0.4941	0.9067	1.1785	0.8609
