# Supplementary material for: Evolutionary patterns in squamate mitogenomes: Are selective regimes associated with fossoriality and limblessness?
Source: Genet Mol Biol. 2026 Jul 20;49(Suppl 2):e20250226. doi: 10.1590/1678-4685-GMB-2025-0226 (PMC13384248; doi:10.1590/1678-4685-GMB-2025-0226)
Supplement: Table S6 - [file 1415-4757-GMB-49-s2-e20250226-s10.pdf]

Genetics and Molecular Biology

https://doi.org/10.1590/1678-4685-GMB-2025-0226

Supplementary Material to "Evolutionary patterns in squamate mitogenomes: are selective regimes associated with fossoriality and limblessness?"

Table S6 – Results of HyPhy's RELAX. Abbreviations: ω = ratio of non-synonymous to synonymous substitutions (dN/dS); np = number of parameters; LnL = log-likelihood; LRT = likelihood ratio test; k = k-value (parameter that quantifies the selection intensity relative to the reference branches).

a.

	LIMBLESS									
		LnL	np	ω				LRT	pval	k
	alternative	-376702.3	127	Reference	N/A	0.80 (1.22%)	9999999171.60 (7.96%)			
ALL				Test	N/A	0.80 (1.22%)	9999999171.60 (7.96%)	21.4	<0.0001	1.00
	null	-376713.0	126	Reference	N/A	0.80 (1.23%)	9999999171.60 (7.95%)			
	alternative	-22492.7	127	Reference	0.00 (78.09%)	0.37 (21.65%)	4270.58 (0.26%)			
ATP6				Test	0.00 (78.09%)	0.58 (21.65%)	89.27 (0.26%)	17.0	<0.0001	0.54
	null	-22501.2	126	Reference	0.00 (76.68%)	0.38 (20.74%)	1.91 (2.58%)			
	alternative	-7097.0	125	Reference	N/A	0.87 (0.33%)	N/A			
ATP8				Test	N/A	1.00 (45.22%)	N/A	0.0	1.0000	1.00
	null	-7097.0	124	Reference	N/A	0.87 (0.33%)	N/A			
	alternative	-35636.1	127	Reference	0.00 (90.71%)	0.14 (8.97%)	7.57 (0.32%)			
COX1				Test	0.00 (90.71%)	0.18 (8.97%)	5.92 (0.32%)	9.8	0.0017	0.88
	null	-35641.0	126	Reference	0.00 (90.92%)	0.17 (8.75%)	6.01 (0.33%)			
	alternative	-17776.1	127	Reference	0.00 (78.31%)	0.14 (21.27%)	16.23 (0.42%)			
COX2				Test	0.00 (78.31%)	0.19 (21.27%)	10.36 (0.42%)	6.8	0.0091	0.84
	null	-17779.5	126	Reference	0.00 (80.79%)	0.19 (18.85%)	22.26 (0.36%)			
	alternative	-20022.9	127	Reference	0.00 (90.15%)	0.52 (9.53%)	9999999171.60 (0.32%)			
COX3				Test	0.00 (90.15%)	0.60 (9.53%)	67313490.42 (0.32%)	2.4	0.1213	0.78
	null	-20024.1	126	Reference	0.00 (90.19%)	0.57 (9.47%)	9999999171.60 (0.34%)			
	alternative	-32448.0	127	Reference	0.00 (87.78%)	0.53 (11.37%)	336.83 (0.85%)			
CYTB				Test	0.01 (87.78%)	0.60 (11.37%)	116.41 (0.85%)	9.0	0.0027	0.82
	null	-32452.5	126	Reference	0.01 (87.14%)	0.53 (11.99%)	238.73 (0.87%)			
	alternative	-26259.5	127	Reference	N/A	0.00 (87.46%)	133.79 (11.97%)			
ND1				Test	N/A	0.00 (87.46%)	81915268.51 (11.97%)	5287.0	<0.0001	3.72
	null	-28903.0	126	Reference	N/A	0.00 (13.28%)	847.03 (3.24%)			
	alternative	-35154.7	127	Reference	0.01 (79.18%)	0.44 (18.42%)	2.53 (2.40%)			
ND2				Test	0.01 (79.18%)	0.44 (18.42%)	2.53 (2.40%)	0.0	1.0000	1.00
	null	-35154.7	126	Reference	0.01 (79.18%)	0.44 (18.42%)	2.53 (2.40%)			
	alternative	-11340.2	123	Reference	0.00 (72.02%)	0.23 (23.13%)	2.13 (4.85%)			
ND3				Test	0.00 (72.02%)	0.30 (23.13%)	1.86 (4.85%)	2.8	0.0943	0.82
	null	-11341.6	122	Reference	N/A	0.23 (25.54%)	1.87 (5.31%)			
	alternative	-44130.5	127	Reference	0.00 (65.80%)	0.10 (24.64%)	1.00 (9.56%)			
ND4				Test	0.00 (65.80%)	0.08 (24.64%)	1.00 (9.56%)	1.0	0.3173	1.06
	null	-44131.0	126	Reference	0.00 (66.26%)	0.09 (24.20%)	1.00 (9.54%)			
	alternative	-10000.6	125	Reference	0.01 (78.13%)	0.43 (21.87%)	N/A			
ND4L				Test	0.02 (78.13%)	0.47 (21.87%)	N/A	0.8	0.3711	0.91
	null	-10001.0	124	Reference	0.02 (78.32%)	0.46 (21.68%)	N/A			
	alternative	-60705.6	127	Reference	0.00 (82.85%)	0.56 (16.54%)	32.65 (0.61%)			
ND5				Test	0.02 (82.85%)	0.70 (16.54%)	8.87 (0.61%)	31.4	<0.0001	0.63
	null	-60721.3	126	Reference	0.01 (77.76%)	0.30 (16.94%)	1.81 (5.30%)			
ND6	alternative	-19166.9	127	Reference	0.00 (62.42%)	0.28 (33.06%)	3.57 (4.52%)			
				Test	0.00 (62.42%)	0.28 (33.06%)	3.57 (4.52%)	0.0	1.0000	1.00
	null	-19166.9	126	Reference	0.00 (62.42%)	0.28 (33.06%)	3.57 (4.52%)			

b.

				FOSSORIAL						
		lnL	np	ω				LRT	pval	k
ALL	alternative	-368873.0	127	Reference	N/A	N/A	9999999171.60 (9.47%)			
				Test	N/A	N/A	9999999171.60 (9.47%)	9.0	0.0027	1.00
	null	-368877.5	126	Reference	N/A	N/A	9999999171.60 (9.47%)			
ATP6	alternative	-22497.2	127	Reference	0.00 (78.31%)	0.43 (20.83%)	2.69 (0.85%)			
				Test	0.00 (78.31%)	0.62 (20.83%)	1.75 (0.85%)	8.0	0.0047	0.56
	null	-22501.2	126	Reference	0.00 (76.60%)	0.38 (20.82%)	1.91 (2.58%)			
ATP8	alternative	-7088.6	125	Reference	0.00 (58.40%)	1.00 (36.43%)	N/A			
				Test	0.04 (58.40%)	1.00 (36.43%)	N/A	4.6	0.0320	0.55
	null	-7090.9	124	Reference	0.00 (55.69%)	0.91 (4.89%)	N/A			
COX1	alternative	-35641.1	127	Reference	0.00 (90.52%)	0.16 (9.14%)	5.87 (0.34%)			
				Test	0.00 (90.52%)	0.16 (9.14%)	5.93 (0.34%)	0.0	1.0000	1.01
	null	-35641.1	126	Reference	0.00 (90.52%)	0.16 (9.14%)	5.87 (0.34%)			
COX2	alternative	-17778.2	127	Reference	0.00 (73.58%)	0.12 (25.06%)	2.10 (1.36%)			
				Test	0.00 (73.58%)	0.10 (25.06%)	2.27 (1.36%)	2.8	0.0943	1.10
	null	-17779.6	126	Reference	N/A	0.10 (27.08%)	2.03 (1.47%)			
COX3	alternative	-20018.6	127	Reference	0.00 (86.10%)	0.16 (9.56%)	1.30 (4.34%)			
				Test	0.00 (86.10%)	0.18 (9.56%)	1.28 (4.34%)	0.2	0.6547	0.93
	null	-20018.7	126	Reference	0.00 (86.14%)	0.17 (9.52%)	1.28 (4.34%)			
CYTB	alternative	-32598.5	127	Reference	0.00 (86.59%)	0.39 (12.06%)	686.60 (1.35%)			
				Test	0.01 (86.59%)	0.66 (12.06%)	18.49 (1.35%)	16.0	0.0001	0.45
	null	-32606.5	126	Reference	0.00 (82.72%)	0.35 (15.86%)	686.60 (1.42%)			
ND1	alternative	-26851.4	127	Reference	N/A	0.19 (24.97%)	2.79 (1.75%)			
				Test	N/A	0.21 (24.97%)	2.60 (1.75%)	1.2	0.2733	0.93
	null	-26852.0	126	Reference	N/A	0.19 (25.66%)	2.75 (1.76%)			
ND2	alternative	-36896.7	127	Reference	N/A	N/A	181.40 (12.51%)			
				Test	N/A	N/A	79083943444.89 (12.51%)	1667.4	0.0000	4.83
	null	-37730.4	126	Reference	N/A	0.25 (9.21%)	471.40 (4.92%)			
ND3	alternative	-11340.3	123	Reference	0.00 (74.91%)	0.30 (20.50%)	1.90 (4.59%)			
				Test	0.01 (74.91%)	0.39 (20.50%)	1.65 (4.59%)	2.6	0.1069	0.78
	null	-11341.6	122	Reference	N/A	0.24 (25.42%)	1.90 (5.16%)			
ND4	alternative	-47025.4	127	Reference	N/A	0.00 (9.49%)	1.86 (7.03%)			
				Test	N/A	0.00 (9.49%)	1.86 (7.03%)	0.0	1.0000	1.00
	null	-47025.4	126	Reference	N/A	0.00 (9.48%)	1.86 (7.03%)			
ND4L	alternative	-10000.9	125	Reference	0.02 (79.66%)	0.48 (20.34%)	N/A			
				Test	0.02 (79.66%)	0.48 (20.34%)	N/A	0.0	1.0000	1.02
	null	-10000.9	124	Reference	0.02 (79.63%)	0.48 (20.37%)	N/A			
ND5	alternative	-60713.2	127	Reference	0.00 (66.65%)	0.11 (24.56%)	1.30 (8.79%)			
				Test	0.00 (66.65%)	0.16 (24.56%)	1.24 (8.79%)	15.0	0.0001	0.82
	null	-60720.7	126	Reference	0.00 (65.39%)	0.13 (26.08%)	1.31 (8.53%)			
ND6	alternative	-19160.0	127	Reference	0.01 (55.24%)	0.08 (31.01%)	1.34 (13.75%)			
				Test	0.01 (55.24%)	0.11 (31.01%)	1.30 (13.75%)	2.8	0.0943	0.89
	null	-19161.4	126	Reference	0.01 (54.88%)	0.09 (30.96%)	1.27 (14.16%)			
